# Myriocin‐induced adaptive laboratory evolution of an industrial strain of *Saccharomyces cerevisiae* reveals its potential to remodel lipid composition and heat tolerance

**DOI:** 10.1111/1751-7915.13555

**Published:** 2020-03-25

**Authors:** Francisca Randez‐Gil, Jose A. Prieto, Alejandro Rodríguez‐Puchades, Josefina Casas, Vicente Sentandreu, Francisco Estruch

**Affiliations:** ^1^ Department of Biotechnology Instituto de Agroquímica y Tecnología de los Alimentos Consejo Superior de Investigaciones Científicas Avda. Agustín Escardino 7 Paterna Valencia 46980 Spain; ^2^ Research Unit on BioActive Molecules (RUBAM) Instituto de Química Avanzada de Cataluña Consejo Superior de Investigaciones Científicas Jordi Girona 18‐26. Barcelona 08034 Spain; ^3^ CIBER‐EHD Instituto de Salud Carlos III Monforte de Lemos 3‐5. Madrid 28029 Spain; ^4^ Genomics Section Central Service for Experimental Research (SCSIE) Universitat de València Dr. Moliner 50 Burjassot 46100 Spain; ^5^ Departament of Biochemistry and Molecular Biology Universitat de València Dr. Moliner 50 Burjassot 46100 Spain

## Abstract

The modification of lipid composition allows cells to adjust membrane biophysical properties in response to changes in environmental temperature. Here, we use adaptive laboratory evolution (ALE) in the presence of myriocin, a sphingolipid (SLs) biosynthesis inhibitor, to remodel the lipid profile of an industrial yeast strain (LH) of *Saccharomyces cerevisiae*. The approach enabled to obtain a heterogeneous population (LHev) of myriocin‐tolerant evolved clones characterized by its growth capacity at high temperature. Myriocin exposure also caused tolerance to soraphen A, an inhibitor of the acetyl‐CoA carboxylase Acc1, the rate‐limiting enzyme in fatty acid de novo production, supporting a change in lipid metabolism during ALE. In line with this, characterization of two randomly selected clones, LH03 and LH09, showed the presence of lipids with increased saturation degree and reduced acyl length. In addition, the clone LH03, which displays the greater improvement in fitness at 40°C, exhibited higher SL content as compared with the parental strain. Analysis of the LH03 and LH09 genomes revealed a loss of chromosomes affecting genes that have a role in fatty acid synthesis and elongation. The link between ploidy level and growth at high temperature was further supported by the analysis of a fully isogenic set of yeast strains with ploidy between 1N and 4N which showed that the loss of genome content provides heat tolerance. Consistent with this, a thermotolerant evolved population (LH40°) generated from the parental LH strain by heat‐driven ALE exhibited a reduction in the chromosome copy number. Thus, our results identify myriocin‐driven evolution as a powerful approach to investigate the mechanisms of acquired thermotolerance and to generate improved strains.

## Graphical abstract

In this work, we have used adaptive laboratory evolution in presence of myriocin, a sphingolipids inhibitor, to remodel the lipid profile of an industrial yeast strain, to investigate the mechanisms of acquired thermotolerance and to generate improved strains.

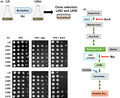

## Introduction

Commercial yeasts are mainly *S. cerevisiae* strains domesticated under artificial selection conditions. They are diploid, triploid, tetraploid and polyploid, and some of them are aneuploids, which is the state characterized by having an abnormal number of certain chromosomes (Sicard and Legras, 2011; Duan *et al.*, 2018). Polyploidization events are frequently associated with the acquisition of broad phenotypic traits, such as robustness, large cell size and high growth rate (Scott *et al.*, 2017), although it could also represent a way for yeast cells to develop phenotypic innovation and adaptation to the various stresses they have to confront in their industrial uses, such as baking, brewing, winemaking or bioethanol production (Fay and Benavides, 2005; Legras *et al.*, 2007; Randez‐Gil *et al.*, 2013; Legras *et al.*, 2018). In addition, aneuploidy may induce genomic instability, as has been recently demonstrated (Sheltzer *et al.*, 2011; Zhu *et al.*, 2012), and thus could facilitate the development of genetic variants. However, this ploidy shift appears to have negative consequences in the behaviour of yeast cells under stress conditions (Randez‐Gil *et al.*, 2013). Large‐scale transitions in genome size from tetraploid or triploid to diploid as the predominant vegetative state of *S. cerevisiae* have been observed during long‐term evolution experiments under stress conditions (Gerstein *et al.*, 2006, 2008; Aguilera *et al.*, 2010; Voordeckers *et al.*, 2015). Thus, the design of domesticated industrial yeasts by polyploidization may not favour the acquisition of stress tolerance phenotypes.

Thermotolerance in yeast is of major importance. Industrial heat‐tolerant yeast strains are currently used for bioethanol production because the fermentations at high temperature (≥ 40°C) facilitate the activity of saccharification enzymes and reduce production costs (Abdel‐Banat *et al.*, 2010). On the other side, the ability of ferment at lower temperature than the optimal is a desired feature of wine and cider yeasts. Cold fermentations reduce the risk of undesirable contaminations during the production process and increase the aromatic complexity of these products (Pérez‐Torrado *et al.*, 2018). Likewise, new species of baker's yeast strains with improved cryoresistance would be also very welcome for frozen dough applications (Randez‐Gil *et al.*, 2013). Moreover, drying to obtain active dried yeast (ADY) or instant dried yeast (IDY; Deák, 2003) imposes extreme heat‐stress conditions on baker's yeast cells that cause cellular damage, leakage of cell constituents and loss of viability. Consequently, manipulation of growth protocols, thermal stress pre‐adaptations and selection of individuals exposed to extreme temperatures during the production processes, has been traditionally used to provide certain thermotolerance. In the practice, the selection and development of cold‐ and heat‐tolerant yeast strains is still a challenge, and the clarification of the genomics of thermal stress tolerance constitute an interesting and promising task.

Previous studies have revealed several mechanisms involved in thermal tolerance acquisition, being likely lipid homoeostasis the most important (Caspeta *et al.*, 2014; Liu *et al.*, 2017). Lipids are essential components of eukaryotic membranes and the main determinants of their functionality as cellular barriers. In yeast, a lower environmental temperature brings about a change in membrane lipid composition, characterized by an increased abundance of phospholipids (PLs) with shorter chain lengths and/or unsaturated fatty acids (Rodríguez‐Vargas *et al.*, 2007). On the contrary, membrane fluidity increases in heat‐stressed yeast cells, which appear to trigger lipid homoeostasis mechanisms to sustain membrane functionality. By using adaptive laboratory evolution (ALE) to generate heat‐stress tolerant evolved clones, Caspeta and colleagues (2014) identified increased sterol accumulation as a mechanism to regulate membrane fluidity and allow improved thermotolerance. Evidence also links the heat‐shock response in *S. cerevisiae* to the activation of sphingolipids (SLs) regulatory networks (Sun *et al.*, 2012), which leads to the rapid and transient accumulation of long‐chain bases (LCBs) and ceramides (Cer) (Dickson *et al.*, 1997; Jenkins *et al.*, 1997), the precursors of complex SLs (Megyeri *et al.*, 2016). Complex SLs with very‐long‐chain fatty acids (VLCFAs) exhibit a higher affinity for sterols, and together with them, promote the formation of a thicker, more compact and less permeable plasma membrane than that provided by a lipid matrix which contains high proportions of unsaturated PLs (Lester *et al.*, 2013).

Yeast adaptation to thermal stress may be also favoured by mutations in genes or molecular pathways involved in membrane organization. Like other eukaryotic cell plasma membranes, the *S. cerevisiae* membrane is asymmetric with an enrichment of phosphatidylserine and phosphatidylethanolamine on the inner leaflet (Muthusamy *et al.*, 2009). This phospholipid asymmetry is established and maintained by lipid translocases or flippases, encoded in yeast by five genes, *DRS2*, *NEO1*, *DNF1*, *DNF2* and *DNF3* (López‐Marqués *et al.*, 2011). Studies in our laboratory (García‐Marqués *et al.*, 2016) and others (Hua *et al.*, 2002; Saito *et al.*, 2004) have identified phospholipid asymmetry as an important aspect that influences cold tolerance by modifying the composition and activity of plasma membrane‐associated proteins. Interestingly, flippase mutants also show resistance to myriocin, a well‐known inhibitor of serine palmitoyltransferase, SPT (Wadsworth *et al.*, 2013), the first enzyme in the *de novo* SL biosynthesis pathway (Megyeri *et al.*, 2016) that enters the cells via the action of flippases (Khakhina *et al.*, 2015).

Here, we have induced genomic changes in an industrial yeast strain of *S. cerevisiae* by ALE in the presence of myriocin. Our hypothesis was that myriocin‐driven evolution could be a suitable strategy to modify the lipid composition and/or asymmetry of the plasma membrane, and as a result, the thermal adaptation of industrial strains. The results presented in this work validate this strategy and add new knowledge on the mechanisms that guide the yeast response to changes in environmental temperature.

## Results and discussion

### Adaptive evolution in the presence of myriocin

**Figure 1:**
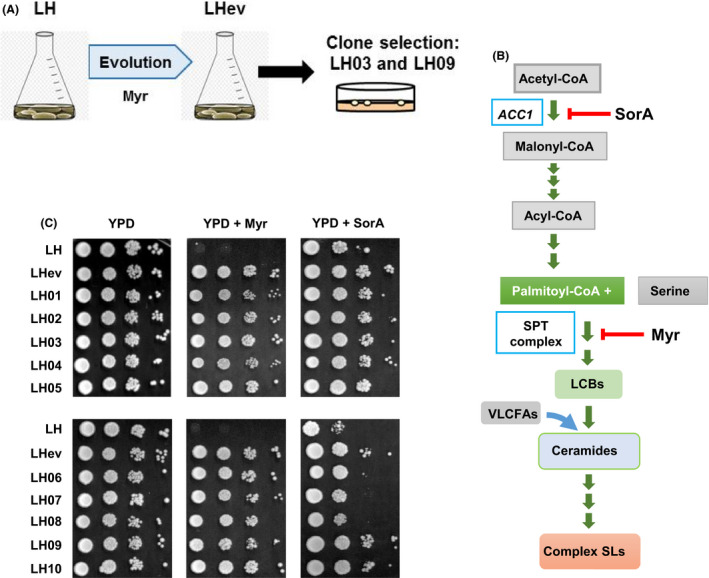
The adaptive evolution experiment, its metabolic context and initial characterization of the evolved population. A. Schematic representation of the myriocin‐driven evolution approach. B. Main metabolic steps involved in the synthesis of complex SLs from acetyl‐CoA. The red arrows indicate the enzymes inhibited by myriocin (SPT) and soraphen A (Acc1). For more details, see text and important reviews (Henry *et al.*, 2012; Klug and Daum, 2014; Huang *et al.*, 2014; Megyeri *et al.*, 2016). C. Growth of the parental strain LH, 50‐generation evolved population LHev and isolated clones LH03 and LH09, was assayed at 30°C in the absence (YPD) or presence of 1.2 μM myriocin (YPD + Myr) or 0.25 μg ml^−1^ soraphen A (YPD + SorA). In all cases, cells were pre‐grown and treated as described in the Material and Methods section.

We used a robust industrial baker's yeast strain named L'Hirondelle (LH) in order to force by ALE changes in the lipid metabolism. For this, yeast was propagated by successive batch refreshments maintained constantly in the presence of myriocin at 30°C during 50 generations (see Fig. 1A). In these experiments, a chronic‐medium dose of 1.2 μM myriocin was used to lower but not completely inhibit SPT activity (Huang *et al.*, 2014; Fig. 1B). Moreover, a short‐term experiment was chosen because deleterious mutations, which could affect negatively important traits of industrial strains, such as cell size, fitness or carbon‐source utilization (Aguilera *et al.*, 2010; Wenger *et al.*, 2011; Çakar *et al.*, 2012; Strucko *et al.*, 2018), have been reported to accumulate over time (Tenaillon *et al.*, 2016). In general, the rates of spontaneous mutation are higher for neutral and deleterious mutations and lower for beneficial mutations (Barrick and Lenski, 2013; Van den Bergh *et al.*, 2018). In this manner, we seek to generate evolved clones containing stable compensatory mutations conferring increased SL biosynthesis (see Fig. 1B) or altered PL asymmetry, but still maintaining key industrial properties. We found that the doubling time decreased gradually over the course of the experiment (data not shown), indicating that the original yeast population was capable of adapting to optimize growth in the presence of the drug. As shown in Fig. 1C, the 50‐generations evolved population (LHev) grew in a solid myriocin‐containing YPD medium, while the parental strain did not. As expected, tolerance to myriocin was also observed for 10 individual adaptive clones isolated from the terminal population. In addition, all of them showed a similar growth (Fig. 1C), suggesting that adaptation‐driving mutations were distributed across the population (Tenaillon *et al.*, 2016).

### The evolved population shows increased tolerance to soraphen A

We tested the growth of the experimental population and individual clones in the presence of soraphen A (Fig. 1C), an inhibitor of acetyl‐CoA carboxylase, ACC (Gerth *et al.*, 1994; Vahlensieck *et al.*, 1994; Gerth *et al.*, 2003; Hofbauer *et al.*, 2014). ACC inhibitors lower malonyl‐CoA content, interfere with fatty acid elongation and alter cellular composition of lipid classes and sub‐classes (Jump *et al.*, 2011; see also Fig. 1B). We reasoned that if myriocin‐directed evolution caused global changes in lipid metabolism, this would be reflected in a change in tolerance to soraphen A. On the contrary, if selection had altered the myriocin uptake mechanisms, resistance to soraphen A would remain unchanged. Myriocin and soraphen A differ in chemical nature, and it may be predicted they use different internalization mechanisms and do not display drug synergy (Yilancioglu *et al.*, 2014). As it is shown, cells of either the terminal experimental population LHev or the individual clones (LH01–10) displayed increased tolerance to soraphen A as compared with that of the original population LH (Fig. 1C). Nevertheless, the improvement in drug tolerance differed among individuals, indicating that the evolution experiment generated phenotypic diversity.

### Myriocin‐driven evolution alters yeast growth at different temperatures

**Figure 2:**
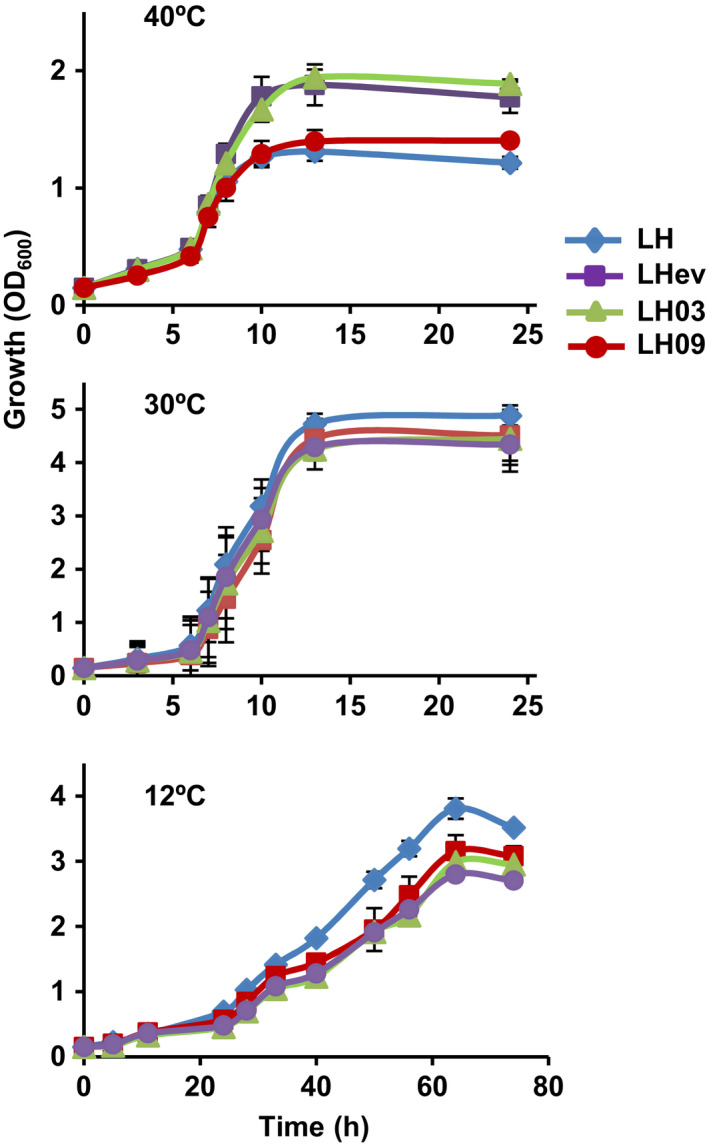
Growth at different temperatures of the evolved populations. Cells of the parental LH strain, evolved population LHev and isolated clones LH00‐LH10 were pre‐grown in liquid SCD medium and refreshed in the same medium (initial OD_600_ ~ 0.5), and their growth at 12, 30 or 40°C was followed for the indicated time. Data represent the mean value (± SD) of three biological replicates.

We asked whether the changes in myriocin tolerance observed in the evolved population were relevant for the physiological response to thermal stress. As shown in Fig. 2, growth at 30°C, the optimal temperature for *S. cerevisiae* of the yeast population was unaffected by the evolutionary experiment. On the contrary, the terminal population exhibited increased growth at 40°C as compared with the parental, whereas a trade‐off in cell proliferation under cold conditions was observed (Fig. 2). Likewise, most of the selected clones under study (LH01–LH10) exhibited increased fitness at 40°C (data not shown) although again the phenotype differed quantitatively among individuals as it is illustrated for two randomly selected clones, LH03 and LH09 (Fig. 2). Thus, the clone LH09 exhibited only a slight growth advantage at 40°C, as compared with the parental LH strain, while the LH03 clone grew much faster and had a similar behaviour to that of the LHev population. Finally, these phenotypes correlated again with a loss in cold growth (Fig. 2). We conclude that myriocin‐drive evolution improved yeasts' ability to adapt to increased temperature with no apparent trade‐offs at the ancestral optimal temperature of 30°C. The results also highlight how tolerance to high temperature is associated with fitness impairments under cold‐stressful conditions.

### SL composition of evolved clones

**Figure 3:**
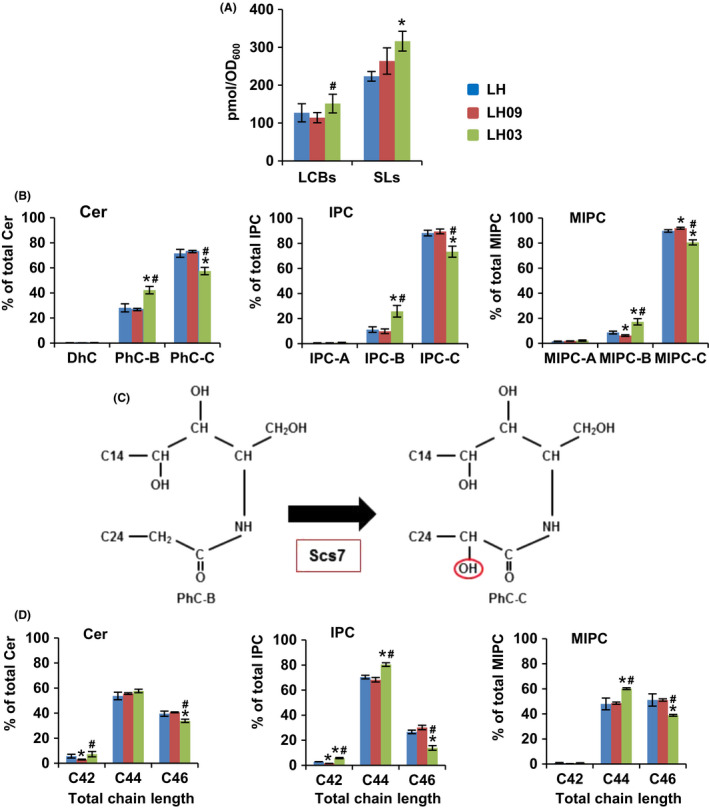
Myriocin‐driven adaptive evolution alters SL composition. A. The relative content of LCBs and SLs in YPD‐grown cells (OD_600_ ~ 1.0) of the LH, LH03 and LH09 strains was analysed by UPLC. Sphinganine (d17:0) and C12 Ceramide (d18:1/12:0) were used as internal standards of LCBs and SLs respectively. The levels of the different lipid species in each sample were normalized to the corresponding internal standard (pmol eq) and units of processed cells (OD_600_). Phosphorylated LCBs were below the detection limit. The quantity of SLs represents the sum of Cer, IPC and MIPC. B. The amount of Cer, IPC and MIPC sub‐classes was normalized (%) to the total content of each class. C. Hydroxylation of phytoceramide (PhC‐B) by Scs7 generates α‐OH‐phytoceramide (PhC‐C). These Cer sub‐classes are then converted to the corresponding IPC‐B and IPC‐C, and subsequently to MIPC‐B and MIPC‐C respectively. For more details, see important reviews (Huang *et al.*, 2014; Megyeri *et al.*, 2016). D. The quantities of Cer, IPC and MIPC species containing the same number of carbon atoms are summed, and these values were normalized (%) to the total amount of the corresponding SL class. In all cases, the data were calculated from at least three biological replicates (± SD). Statistically significant differences (*P* < 0.05) between LH and LH03 or LH09 samples (*), and between the evolved clones LH03 and LH09 (#) are indicated.

We analysed more in deep whether our evolutionary experiment forced a change in the lipid metabolism of yeast cells. The SL profile of the evolved clones LH03 and LH09 was analysed by LC‐MS/MS and the results compared with those of the parental LH strain. As shown in Fig. 3A, the evolved clone LH03 showed a higher content of SLs than the parental LH strain, while the changes in LCBs or in the amount of SLs of the clone LH09 were not statistically significant. We also observed a distinct behaviour within the SL sub‐classes of the LH03 strain (Fig. 3B). Indeed, the relative abundance of Cer‐B, IPC‐B and MIPC‐B increased in cells of this strain, while that of the serie‐C members decreased (Fig. 3B). This was also evident in the major species of each sub‐class (Supplementary Tables S1–S3). The classical yeast SL nomenclature stablishes five sub‐classes of Cer, IPC, MIPC and M(IP)_2_C; A, B, B', C and D, according to their hydroxylation degree (Megyeri *et al.*, 2016). In our analysis, species from the serie‐B and B', which contain both three OH groups, are indistinguishable, and those of the serie‐D cannot be determined. Molecules of the serie‐C (four OH) are formed from those of the serie‐B by hydroxylation catalysed by the enzyme Scs7 (Haak *et al.*, 1997; see Fig. 3C). Finally, we also observed a significant increase in C42‐species of Cer and C44‐species of IPC and MIPC, and a parallel decrease in those of C46 in the LH03 strain (Fig. 3D). Since we used a mass spectrometry protocol without fragmentation, we cannot distinguish between the contribution of the LCB and the acyl group to the total chain length. Nevertheless, it is clear that the evolved clone LH03 forms SLs with statistically shorter chain length.

### Myriocin‐dependent regulation of neutral lipids and phospholipids

**Figure 4:**
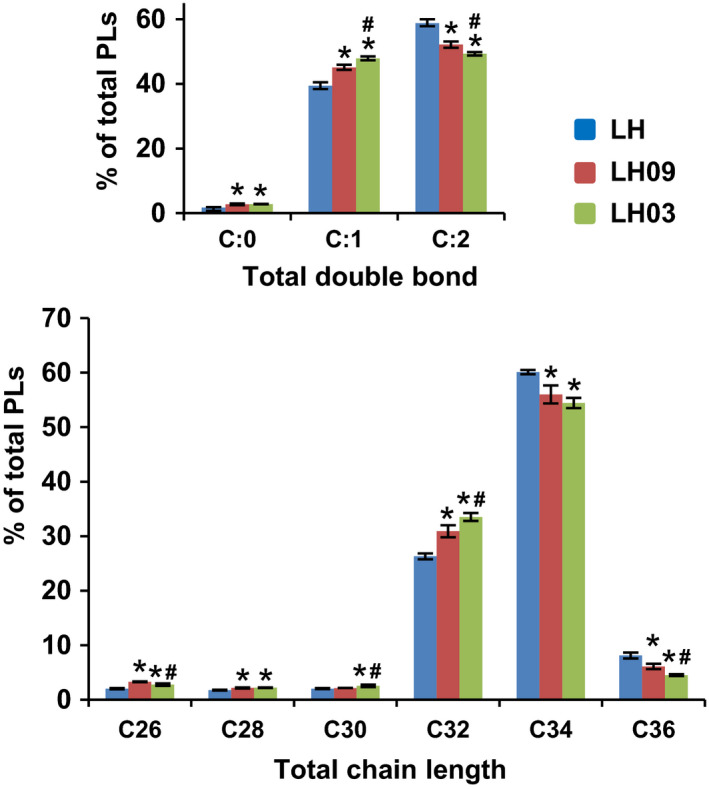
The total acyl chain length and the unsaturation degree of PLs are affected in response to chronic myriocin exposure. The quantities of the PL species containing the same number of double bonds or the same number of carbon atoms in the hydrocarbon moiety are summed, and these values were normalized to the total amount of PLs (%). Statistically significant differences (*P* < 0.05) between LH and LH03 or LH09 samples (*), and between the evolved clones LH03 and LH09 (#) are indicated. Data represent the mean value (± SD) of three independent biological replicates.

Lipid metabolic pathways are highly interconnected in order to obtain a balanced composition of classes and sub‐classes of lipids. Accordingly, we examined the type and amount of neutral lipids (NLs) and PLs in the evolved clones under study. Compared with the parental strain, the mutant strains analysed did not show significant changes in their absolute content of NLs and PLs (Fig. S1). Neither their relative abundance (mol%) of steryl esters (SE), diacylglycerol (DAG), triacylglycerol (TGA), phosphatidate (PA), phosphatidylcholine (PC), phosphatidylethanolamine (PE), phosphatidylglycerol (PG), phosphatidylinositol (PI) and phosphatidylserine (PS) showed apparent variations (Fig. S1). However, the composition of molecular species revealed noticeable changes (Tables S4–S12), affecting mainly chain length and insaturation degree. In general, PLs (Fig. 4 and Tables S7–S12) and NLs (Tables S4–S6) species with shorter chain length and increasing saturation state became more abundant in response to chronic myriocin exposure. Unlike the SL data showed above, similar results were observed for both evolved clones, LH09 and LH03, although the changes were quantitatively more pronounced for LH03 cells. Such changes could have a great impact in membrane properties, and thus, they could affect the adaptive response to thermal stress and influence the phenotypic traits of the evolved population. For example, increased SL content and lower acyl desaturation could be expected to rigidify the plasma membrane, and thus compensating for enhanced membrane fluidity at high temperature. However, shorter acyl backbones have the opposite effect. Hence, the attenuation of fatty acid elongation in the evolved clones could be mainly a determinant not of heat tolerance but of increased resistance to myriocin toxicity.

### Phenotypic characterization of *elo* mutants

**Figure 5:**
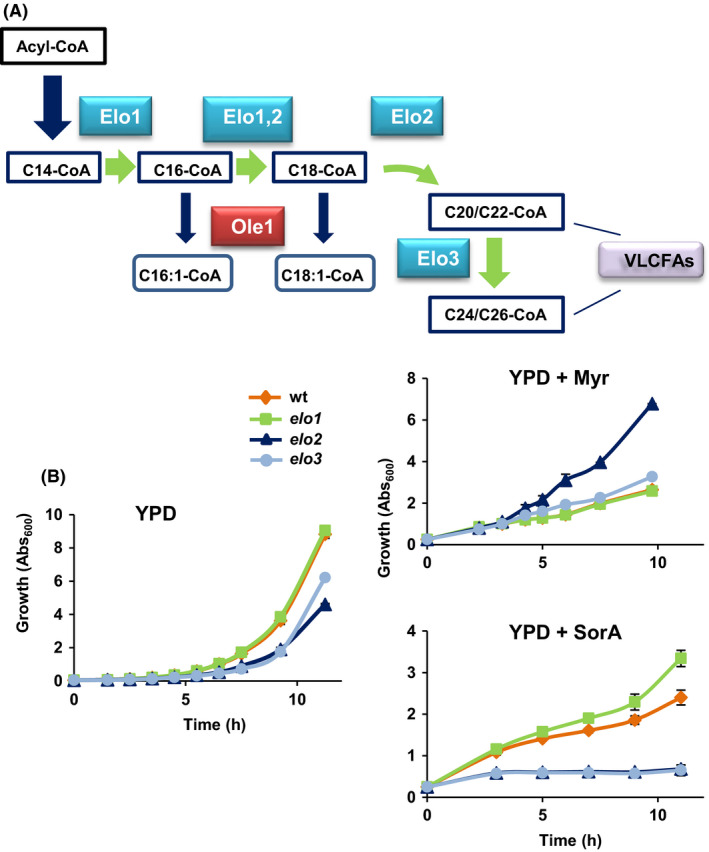
Impaired fatty acid elongation affects growth of yeast cells on myriocin and soraphen A. A. Schematic representation of fatty acid elongation and desaturation in the yeast endoplasmic reticulum (ER). The reactions scheme up to C26 and the elongases involved, Elo1, Elo2 and Elo3 are shown. Fatty acids are monounsaturated via a reaction catalysed by the ER‐resident and essential Δ9 desaturase, Ole1. For more details, see representative reviews (Tehlivets *et al.*, 2007; Henry *et al.*, 2012; Klug and Daum, 2014; Huang *et al.*, 2014; Megyeri *et al.*, 2016). B. Cells of the laboratory wild‐type BY4741 strain (wt) and its corresponding elongase mutants, *elo1*, *elo2* and *elo3*, were pre‐grown in liquid YPD medium and refreshed in the same medium (initial OD_600_ ~ 0.05) lacking (YPD) or containing 2.0 μM myriocin (YPD + Myr) or 0.25 μg ml^−1^ soraphen A (YPD + SorA), and their growth was followed for the indicated time. Data represent the mean value (± SD) of three biological replicates.

The above results identified the fatty acid elongation as one of the lipid metabolic processes altered in both LH03 and LH09 by the 50‐generation myriocin exposure. The products of three genes in *S. cerevisiae* have an important role in this process, *ELO1*, *ELO2* and *ELO3,* which take place in the endoplasmic reticulum (Tehlivets *et al.*, 2007). The elongase Elo1 extends C12‐C16 fatty acyl‐CoAs to C16‐C18 fatty acids (Toke and Martin, 1996), while Elo2 elongates palmitoyl‐CoA and stearoyl‐CoA up to C22 fatty acids, and Elo3 produces C20‐C26 VLCFAs, which form part of Cer and complex SLs (Megyeri *et al.*, 2016), from C18‐CoA primers (Oh *et al.*, 1997; Rössler *et al.*, 2003). Figure 5A shows a schematic representation of the fatty acid elongation process in yeast cells. Thus, changes in Elo1 activity may have effects in acyl constituents of all lipid classes, SLs, NLs or PLs, while those in Elo2 and Elo3 could alter mainly the SL composition. According to this, we first examined the growth of the *elo1*, *elo2* and *elo3* mutants of the laboratory wild‐type strain BY4741 in liquid YPD medium containing or lacking myriocin and soraphen A. As shown in Fig. 5B, knock‐out of *ELO2* or *ELO3* caused a clear defect in the growth of wild‐type cells in YPD control medium at 30°C, while no significant effect could be detected by the absence of Elo1. Despite this, cells devoid of Elo2 activity grew much better than the wild type in the presence of myriocin (Fig. 5B), a result previously reported by Olson and colleagues (2015) in a different yeast background. Furthermore, the lack of Elo3 provided a statistically significant growth advantage, but the effect was scarce, while no effect could be detected in *elo1* mutant cells (Fig. 5B). On the other hand, cells lacking the elongase activity provided by Elo2 or Elo3 were unable to grow in soraphen A‐containing medium, suggesting that the synthesis of Cer and/or complex SLs could be critical when cells are exposed to Acc1 inhibitors. On the contrary, mutation of *ELO1* rendered yeast cells more tolerant to soraphen A (Fig. 5B).

We then analysed the growth of the mutants at 40°C (Fig. S2). Again, *elo2* and *elo3* strains showed, as compared with the wild type, a growth defect similar to that observed at the optimal 30°C temperature (Fig. 5B; YPD). Neither the loss of Elo1 caused an apparent advantage under high‐temperature conditions (Fig. S2). Finally, we checked the phenotypes of cells lacking the hydroxylase Scs7. We were unable to observe increased growth in the presence of myriocin or aureobasidin A (data not shown), or in response to thermal stress (Fig. S2). Taken together, these results suggest that a reduced elongase activity might be a determinant of the myriocin‐ and soraphen A‐tolerance phenotype exhibited by the industrial evolved clones. The relationship between this activity and heat tolerance is, however, unclear.

### Adaptive laboratory evolution causes aneuploidy and loss of ELO gene copy number

The emergence of aneuploidy, including whole‐chromosome loss, in response to different environmental and stressful conditions has been widely observed in a wide range of organisms (Ben‐David and Amon, 2020), including *S. cerevisiae* (Gerstein *et al.*, 2006, 2008; Aguilera *et al.*, 2010; Pavelka *et al.*, 2010; Voordeckers *et al.*, 2015; Selmecki *et al.*, 2015). Accordingly, we analysed the genomes of the two evolved clones isolated from the industrial LH strain. First, we examined the DNA content by flow cytometry (Fig. S3). As we expected, the ploidy of the parental and the evolved populations was higher than that of the laboratory strains used as control, BY4741 (1N, haploid) and BY4743 (2N, diploid). Interestingly, the DNA content per cell decreased in both evolved strains after 50 generations of myriocin exposure. Thus, the estimated average chromosome number for the parental strain (LH) was 4.1, whereas for LH03 was 3.0 and 3.6 for LH09. This reduction was confirmed by the chromosome copy number predictions from the sequencing data (Table 1). Indeed, the likelihood ploidy for the sixteen chromosomes of the parental strain was tetraploid, while there was a reduction in the number of copies in nine of the chromosomes of strain LH03 and in three of the chromosomes of strain LH09.

**Table 1:** Likelihood ploidy by chromosome in the parental and evolved strains.

Chr.	Gene^a^	Likelihood ploidy		
		LH	LH03	LH09
I		tetra	di	tri
II		tetra	tetra	tetra
III	*ELO2*	tetra	tri	tetra
IV		tetra	tri	tri
V	*FAA2*	tetra	tri	tri
VI		tetra	tetra	tetra
VII	*ACB1*	tetra	tetra	tetra
VIII		tetra	tetra	tetra
IX	*FAA3*	tetra	tetra	tetra
X	*ELO1*	tetra	tri	tetra
XI	*FAS1*	tetra	tetra	tetra
XII	*ELO3*	tetra	tri	tetra
XIII	*SCS7*, *FAA4*	tetra	tri	tetra
XIV		tetra	tri	tetra
XV	*FAA1*	tetra	tetra	tetra
XVI	*FAS2*	tetra	tri	tetra

^a^ Relevant lipid metabolism gene included in this chromosome.

Then, we check whether the ploidy reduction observed in the evolved strains altered the lipid‐gene doses, in particular of those whose activity may influence the acyl chain length (Table 1). Indeed, we found that LH03 cells lost a copy of the chromosomes III, X and XII, where the genes *ELO2*, *ELO1* and *ELO3* are located respectively (Table 1). In addition, the myriocin‐evolved approach resulted in a reduction in the copy number of genes involved in fatty acid activation (*FAA2* and *FAA4*) and synthesis (*FAS2*), which could contribute to the changes observed in the acyl chain length of NLs and PLs of both, LH03 and LH09 (Fig. 4 and Tables S4–S12). Finally, we also noted that cells of the clone LH03, but not those of the clone LH09, lost a copy of the hydroxylase‐encoding gene *SCS7* (Table 1; chromosome XIII), which could explain the decrease in the relative abundance of SL species of the serie‐C found in the former (Fig. 3B).

### Point mutation analysis

The whole‐genome sequencing data were further analysed in search of single‐nucleotide polymorphisms (SNPs). The identification of point mutations occurred during evolution proved to be difficult because the elevated ploidy of the strains and the chromosome copy number variation observed. For these reasons, the SNP calling was settled on an approach that required > 15% base‐call supporting a SNP in the evolved genomes and < 5% base‐call supporting the same base in the parental genome data and genotype quality above 20. Applying this approach, we detected 78 single point mutations found only in the evolved LH03 strain (Table S13), whereas 10 SNPs were only found in the evolved LH09 strain (Table S14) and 28 were found in both evolved strains (Table S15). The most streaking result was observed in the SNPs detected in the LH03 strain. Most of the polymorphisms found in coding regions were located in ribosomal protein coding genes (45 out of a total of 60). Even more striking is that all the changes identified give synonymous variants. A detailed analysis of the amino acids affected by these changes reveals that they are mostly non‐polar (31 of 45) and that the changes give rise to triplets with a lower codon usage value (26 SNPs increase this value, while 15 reduce it). These changes could lead to the attenuation of the levels of ribosomal protein translation, which in turn might improve the fitness of the aneuploid evolved clones by reducing proteotoxic stress. Evidence of a link between protein synthesis rate and several phenotypes shared by all aneuploidy human cells has been reported (Torres *et al.*, 2007; Oromendia *et al.*, 2012). On the other hand, SLs are synthesized from serine and palmitoyl‐CoA (Megyeri *et al.*, 2016), and consequently reduced protein translation could increase the availability of this amino acid and the levels of SLs as it was observed in the LH03 strain (Fig. 3A).

In relation to the SNPs identified exclusively in strain LH09 or that are common in both evolved strains, we find remarkable the N370D polymorphism (present in both strains) that affects the *CST26* gene, encoding an acyltransferase enzyme responsible for the introduction of saturated very‐long‐chain fatty acids into phosphatidylinositol (Le Guédard *et al.*, 2009). Interestingly, this gene was initially identified in a search for genes that affect chromosome stability (CST) when overexpressed in *S. cerevisiae* (Ouspenski *et al.*, 1999). Hence, the changes identified in this gene may be associated with the genomic instability and increased chromosome loss displayed by the evolved clones.

### Loss of ploidy level drives adaptation to heat stress

**Figure 6:**
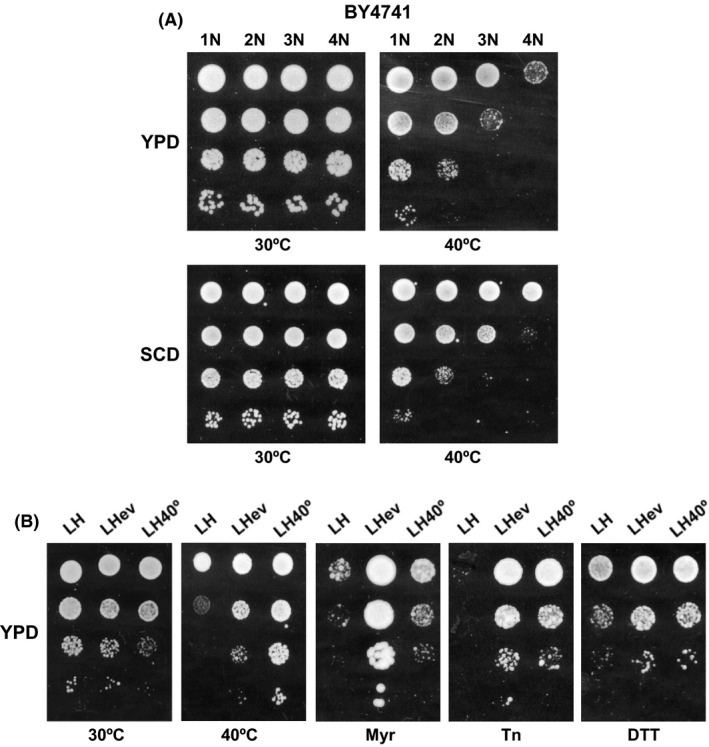
Ploidy effects and phenotypic variation of myriocin‐ and heat‐evolved terminal populations. A. A serie of isogenic BY4741‐derivative strains differing in ploidy level (1N, 2N, 3N and 4N; Storchová *et al.*, 2006) were analysed for growth. Tenfold serial dilutions of saturated cultures grown in minimal SCD or rich YPD medium were prepared, and 3 μl aliquots of three dilutions (10–103) were applied over SCD‐ or YPD agar‐gelled plates. Colony growth was inspected after 2 days of incubation at 30°C or 40°C. B. Growth of the myriocin (LHev)‐ and heat‐evolved (LH40°) terminal populations of the industrial parental strain L'Hirondelle (LH) was assayed at 40°C or at 30°C in the absence (YPD) or presence of 0.75 μM myriocin (Myr), 0.4 mM tunicamycin (Tn) or 5 μM DTT (DTT). In all cases, cells were pre‐grown at 30°C or 40°C and treated as described above. In all cases, a representative experiment is shown.

To gain insight into how chromosome loss affects the phenotypic characteristics of the LHev evolved population, we first compared the growth at high temperature of an isogenic series of diploid (2N), triploid (3N) and tetraploid (4N) yeasts generated from the haploid (1N) laboratory strain BY4741 (Storchová *et al.*, 2006). As it is shown in Fig. 6A, loss of chromosome copy number provided a fitness advantage to yeast cells grown at 40°C in either YPD or minimal SCD medium, while no effect was observed at the optimal 30°C temperature. Quite remarkably, the increased growth was correlated with the loss of ploidy level ranking from 4N to 1N (Fig. 6A). Next, we compared the growth under different environmental conditions, such as H_2_O_2_‐mediated oxidative stress, hyperosmotic NaCl or presence of tunicamycin, which induces endoplasmic reticulum stress. Under all these conditions, the analysed strains grew similarly (Fig. S4). Neither the ploidy level had a noticeable effect on the growth of yeast cells in SCD culture medium containing myriocin, aureobasidin or soraphen A (Fig. S4). Our results indicate a strong relevance of ploidy level in the context of thermotolerance, an effect that could be related to proteotoxicity. It has been reported that yeast strains harbouring an additional copy of a single yeast chromosome, called disomes, display a higher load of endogenous protein aggregates and exhibit increased sensitivity to high temperature (Oromendia *et al.*, 2012). In addition, proteotoxicity is also a hallmark of heat stress (Joutsen *et al.*, 2020). However, the fact that polyploidy did not alter growth in the presence of tunicamycin (a condition that also requires the assistance of the cell's protein quality control pathways; Fig. S4) is indicative of the complex relationship between thermal stress and ploidy.

Finally, we wonder whether adaptation to high temperature in the industrial strain selects cells with decreased ploidy level. Like the myriocin‐driven experiment, cells of the parental yeast LH were propagated by successive batch refreshments maintained constantly at 40°C during 50 generations and the DNA content of the evolved population (LH40°) was examined by flow cytometry. As expected, heat exposure promoted a loss of chromosome copy number in the heat‐exposed terminal population (2.9N). Similar decreases were also observed for isolated clones from LH40° (data not shown).

### Heat‐ and myriocin‐evolved populations display common and distinct phenotypes

We wonder if our initial choice of myriocin as evolutionary condition to select, among others, thermotolerant strains was appropriate in terms of biotechnological suitability. In this context, the best phenotype is not always necessarily the one with the highest fitness, but the one that shows increased performance and the least trade‐offs in other environmental conditions (Dragosits and Mattanovich, 2013). Thus, we compared the phenotypic profile of heat‐ and myriocin‐evolved industrial yeast cells. As expected, the heat‐evolved population exhibited higher fitness at 40°C than the LHev, and this late grew much faster than the LH40° in the presence of myriocin (Fig. 6B). Furthermore, both evolved populations exhibited increased tolerance to H_2_O_2_ (data not shown) or to either tunicamycin or DTT (Fig. 6B), indicating likely cross‐protection (Dragosits and Mattanovich, 2013). More importantly, the cells that were evolved under heat show an evident growth trade‐off at the optimal growth temperature for yeast (Fig. 6B; 30°C). Based on these data, the myriocin approach appears as an interesting strategy to isolate industrially relevant yeast strains.

## Concluding remarks

Our study points out the importance of downregulating the fatty acid elongation as a mechanism to face the SL biosynthesis inhibition. The key players of this regulation were the reticulum endoplasmic elongases Elo1, Elo2 and Elo3, although impaired cytosolic fatty acid synthase activity may be on the basis of the shorter fatty acids exhibited by NLs and PLs in the clone LH09. The activity of all these enzymes involves the condensation/decarboxylation of acyl‐CoA with Acc1‐derived malonyl‐CoA (Tehlivets *et al.*, 2007). Thus, decreased elongase activity reduces the demand of malonyl‐CoA and increases the availability of palmitoyl‐CoA, the precursor together with serine of LCBs, alleviating likely the myriocin‐instigated inhibition of SPT. Consistent with this mechanism, the evolved clones showed increased resistance to soraphen A, the Acc1 inhibitor, and displayed lower acyl desaturation degree. This suggests the changes in saturation were likely addressed to provide an additional supply of palmitoyl‐CoA. Altogether, the findings underline the crosstalk between lipid metabolic pathways, and the close interaction of fatty acid elongation and desaturation with SL metabolism.

Biochemical characterization of the evolved clones revealed differences that could explain their phenotypic variation. Our results suggest that α‐hydroxylation of Cer, IPC and MIPC, in combination with the higher content of SLs, could play a role in the acquired thermotolerance mechanism observed in the LH03 clone. It is well known that heat‐shock increases the yeast SL content, contributing, together with sterols, to reduce membrane fluidity at high temperature. The rationale to replace SLs of the serie‐C by those of the serie‐B in that clone is less obvious, although it may be related to toxicity differences between Cer sub‐classes and species. It has been suggested that PhS‐based Cer are more toxic than DhS‐based ones, while the acyl α‐hydroxylation level seems to reduce the toxicity of these compounds (Tani and Kuge, 2012). Hence, the change in the profile of Cer could help to reduce the toxicity produced by elevated Cer levels. Nevertheless, these changes could also play a role in heat‐signalling or mediate the regulation of structural properties of the plasma membrane at high temperature. More work is required to clarify the physiological significance of these changes.

The myriocin‐driven ALE approach selected evolved clones with a specific and complex aneuploidy pattern. It has been proposed that aneuploidy generates stoichiometric imbalance of protein complexes leading to proteotoxicity and reduced fitness under non‐stress conditions (Pavelka *et al.*, 2010; Chen *et al.*, 2019). However, the evolved clones selected do not show major growth impairment at the ancestral optimal growth temperature (Fig. 2; 30°C). In addition, the loss of chromosome copy number provided a proliferative advantage to myriocin‐ and heat‐exposed cells. SL levels are tightly linked to serine availability, which it is favoured by decreasing the number of encoding genes (Torres, 2015; Hwang *et al.*, 2017), but more importantly, decreasing ploidy level provided additional mechanisms of cell protection at high temperature, that were confirmed by analysis of isogenic strains of BY4741 and heat‐driven evolved yeast populations. Whether this general effect is mediated by decreasing proteotoxicity needs to be investigated.

Taken together, our evolutionary experiment demonstrates that is possible to obtain phenotypic variation and growth advantages under industrially relevant conditions that do not inevitably result in important fitness drawbacks in the optimal environment. Hence, the isolated clones in our study are of great value to gain insights about the mechanisms of yeast adaptation and the seed of more robust stress‐resistant strains.

## Experimental procedures

### Yeast strains and cultivation conditions

The *S. cerevisiae* strain L'Hirondelle (LH), a commercial baker's yeast produced by the Lesaffre Group (Lille, France), was used in the evolutionary experiments reported in this study. The laboratory *S. cerevisiae* strains BY4743 (diploid, 2N), BY4741 (haploid, 1N), BY4741‐mutants *elo1*, *elo2*, *elo3* and *scs7*, all of them from the Euroscarf yeast collection (Oberursel, Germany), as well as a serie of BY4741‐derived isogenic strains differing in ploidy level (2N, 3N and 4N; Storchová *et al.*, 2006) were also used. Cells were regularly maintained on solid YPD (20 g l^−1^ agar, 20 g l^−1^ peptone, 20 g l^−1^ glucose and 10 g l^−1^ yeast extract) or SCD (20 g l^−1^ agar, 5 g l^−1^ ammonium sulfate, 20 g l^−1^ glucose and 1.7 g l^−1^ yeast extract without amino acids and ammonium sulfate) supplemented with the appropriate amino acid dropout mixture (Formedium, England).

For plate phenotype experiments, cells were grown to the mid‐exponential phase at 30°C (OD_600_ ~ 0.5). Then, 10‐fold serial dilutions were prepared and 3 μl aliquots of three dilutions (10–10^3^) were applied over the agar‐gelled plates. Colony growth was inspected after 2–4 days of incubation at 12, 30 or 40°C. In some experiments, the growth under stressful conditions was followed in liquid medium. In this case, 30°C‐grown saturated YPD cultures were diluted in the appropriate medium at initial OD_600_ ~ 0.025–0.05. Stock solutions of 1.0 mg/ml (methanol) soraphen A (a gift from R. Jansen) and 2 mM myriocin (ethanol:DMSO; 80:20, v:v) were prepared, sampled in small volumes and stored at −20°C until use. For each experiment, a fresh sample was thawed and diluted at the indicated concentration.

### Laboratory adaptive evolution

Evolution experiments were conducted using batch culture techniques in a similar manner than that described by Aguilera and colleagues (2010). Fifty milliliters of medium YPD containing 1.2 μM myriocin (final concentration) was inoculated at OD_600_ ~ 0.05 and cultured in 250 ml Erlenmeyer flasks at 30°C and 200 rpm. The culture was refreshed daily and cultivated in the same way until a minimum of ~ 50 generations was attained. Heat‐driven ALE was conducted similarly except that cells were grown at 40°C in the absence of myriocin. As each transfer allowed ~ 6.64 mitotic divisions, a total of ~ 8 transfers were carried out. Samples from the evolved populations were taken, maintained as frozen (−80°C) glycerol stocks and then rescued in YPD agar plates at 30°C for 24 h before further analysis. These conditions preserved the characteristics of the evolved population and ensured that yeast cells were growing under the same conditions.

For the selection of adaptive clones within the myriocin‐driven 50‐generation experimental population (LHev), a diluted sample was cultivated in solid YPD containing 2.0 μM myriocin. Then, 10 cells that formed the biggest colonies, LH01–LH10, were randomly selected and preserved at −80°C for further characterization. Individual clones from the terminal heat‐evolved population (LH40°) were also isolated and preserved under the same conditions.

### SL extraction and mass spectrometry analysis

YPD‐grown cells of the parental LH strain and the myriocin‐evolved clones LH03 and LH09 (OD_600_ ~ 1.0) were collected by centrifugation (3000 × *g*, 2 min, 4°C), washed twice with ultrapure water (Milli‐Q RO 10 Plus; Millipore, Bedford, MA, USA) and kept at −80°C until the extraction was carried out. Then, the cell pellet was suspended in 300 μl of ultrapure water, glass beads (1.0 g; acid washed, 0.4‐mm diameter) were added, and the mixture was vortexed 5 times for 1 min each time. Finally, the homogeneous broken cell suspension was recovered, diluted with ultrapure water (~ 20 OD_600_ units/ml) and subjected to SL analysis.

SLs were extracted according to a previous study (Barbacini *et al.*, 2019), with minor modifications. Methanol (1 ml) and chloroform (0.5 ml) were added to 0.5 ml of the cell suspension, fortified with internal standards [200 pmol: sphinganine (d17:0), sphinganine‐1‐phosphate (d17:0), and C12 Ceramide (d18:1/12:0)], vortexed and incubated at 48°C overnight. Then, KOH in methanol (1 M, 75 µl) was added and incubated for 2 h at 37°C. Finally, acetic acid (1 M, 75 µl) was added and samples were evaporated and stored at −80°C. Methanol (80 µl) was added to the samples, vortexed, centrifuged at 9400 × *g* for 5 min and transferred into UPLC vials for their analyses.

Cer, IPC and MIPC analyses were performed using an Acquity ultra‐high performance liquid chromatography (UHPLC) system (Waters) connected to a Time of Flight (LCT Premier XE) detector. Full scan spectra from 50 to 1800 Da were acquired, and individual spectra were summed to produce data points each of 0.2 s. Mass accuracy at a resolving power of 10 000 and reproducibility were maintained by using an independent reference spray via the LockSpray interference. Lipid extracts were injected onto an Acquity UHPLC BEH C8 column (1.7 µm particle size, 100 mm × 2.1 mm, Waters) at a flow rate of 0.3 ml min^−1^ and column temperature of 30°C. The mobile phases were methanol with 2 mM ammonium formate and 0.2% formic acid (A)/water with 2 mM ammonium formate and 0.2% formic acid (B). A linear gradient was programmed. The gradient starts with 80% A and increases to 90% A in 3 min. After 3 min at 90% A, the gradient increases to 99% A in 9 min and continues at 99% A for 3 min. During the following 2 min, the column is readjusted to the initial conditions and equilibrated for further 2 min. Phytosphingosine and phytosphingosine‐1‐phosphate were analysed using an Acquity ultra‐high performance liquid chromatography (UHPLC) system (Waters) connected to a triple quadrupole (Xevo‐TQ‐S) detector. The following transitions were used as follows: 288.3 > 252.3 (C17 dihydrosphingosines), 302.3 > 284.4 (dihydrosphingosines), 318.3 > 282.4 (phytosphingosines), 382.4 > 284.4 (dihydrosphingosines‐1‐P), 398.4 > 282.4 (phytosphingosines‐1‐P). The same column, mobile phases and gradient were used. The levels of the different lipid classes and species in each sample were normalized as pmol eq/OD_600_. All the data were calculated from three biological replicates (± SD).

### NL and PL analysis

Cell samples were processed as described above for SL extraction and subjected to basic lipidomic analysis. This was carried out by the lipidomic service of Lipotype (Lipotype GmbH, Dresden, Germany), which use a high‐throughput mass spectrometry‐based shotgun lipidomics platform. Lipids were extracted using chloroform and methanol (Klose *et al.*, 2012). Samples were spiked with lipid class‐specific internal standards prior to extraction. After drying and resuspending in MS acquisition mixture, lipid extracts were subjected to mass spectrometric analysis. Mass spectra were acquired on a hybrid quadrupole/Orbitrap mass spectrometer equipped with an automated nano flow electrospray ion source in both positive and negative ion modes. Lipid identification using lipotypexplorer (Herzog *et al.*, 2011) was performed on unprocessed (*.raw format) mass spectra. For MS‐only mode, lipid identification was based on the molecular masses of the intact molecules. MSMS mode included the collision‐induced fragmentation of lipid molecules, and lipid identification was based on both the intact masses and the masses of the fragments. Prior to normalization and further statistical analysis, lipid identifications were filtered according to mass accuracy, occupation threshold, noise and background. Lists of identified lipids and their intensities were stored in a database optimized for the particular structure inherent to lipidomic data sets. Intensity of lipid class‐specific internal standards was used for lipid quantification. The amounts of individual lipid molecules (species) of a given lipid class were normalized as the mol%. Likewise, the quantities of the lipid species containing the same number of double bonds or the same number of carbon atoms in the hydrocarbon moiety are summed and these values are normalized to the total amount of the given lipid class. Additional details concerning sample handling and processing, reagents, equipment, procedures and data visualization tools can be found at https://www.lipotype.com/. Data represent the mean value (± SD) of three independent biological replicates.

### Genome size

Flow cytometric analysis was used to estimate the approximate genome size of the industrial yeast strains. Cells were grown in liquid YPD to exponential phase, harvested, washed and fixed in 70% ethanol at 4°C for 5 min. Then, cells were collected by centrifugation and resuspended in 10 mM PBS buffer (pH = 7.2), containing 400 μl of RNase (10 mg ml^−1^). After incubation at 37°C for 30 min, the cells were harvested by centrifugation and resuspended in 1 ml of the same buffer containing 2.5 mg l^−1^ of propidium iodine. Samples were analysed using a flow cytometer FAC Scan analyser (Becton Dickinson, Franklin Lakes, NJ). Ploidy determinations were done by comparing with the laboratory strains BY4741 (1N) and BY4743 (2N).

### Sequencing and bioinformatics analysis

Whole‐genome sequencing and bioinformatics analysis were performed at the Genomics service of the Valencia University (Valencia, Spain). Briefly, Illumina sequencing libraries from LH, LH03 and LH09 strains were constructed using the TruSeq nano DNA library preparation kit (Illumina, San Diego, CA, USA). The number of raw pair‐end 301 bp reads collected was 6 341 808 (LH), 2 594 029 (LH03) and 7 264 515 (LH09). Raw reads were quality trimming and filtering using AfterQC (Chen *et al.*, 2017), with filter of minimum phred‐quality score 15 and minimum read size as 50. Raw and processed read quality control was made with fastqc v0.11.8 (http://www.bioinformatics.babraham.ac.uk) and AfterQC tools. Resulting reads, 5 788 893 (LH), 2 413 082 (LH03) and 6 788 094 (LH09) were aligned to the *S. cerevisiae* R64‐1‐1 reference strain using the bowtie2 mapping tool (Langmead and Salzberg, 2012). samtools 1.19 and picard 2.18 (Li *et al.*, 2009) were used for mapping post‐processing and remove duplicates. Only proper paired reads with a mapping quality score above 30 were retained from the alignment. Indel realignment and depth of coverage calculation were performed with GATK‐3.6.

Alignment data files were quality check with qualimap v2.2.1 (García‐Alcalde *et al.*, 2012; Okonechnikov *et al.*, 2016). Percentage of reference bases covered above four reads were 93.2% (LH), 93.1% (LH03) and 93.2% (LH09) with an average read‐depth of 171.75 (LH), 85.24 (LH03) and 228.76 (LH09). For SNP calling (SNPs and indels detection) and filtering, we use VarScan (v2.3.9; –min‐avg‐qual 20 –min‐var‐frequation 0.05 –min‐coverage 30 ‐min‐reads2 5 –min‐freq‐for‐hom 0.95 –*P*‐value 0.05). MiModD 0.1.9 tool (http://doi.org/10.5281/zenodo.2582000) was used for variant post‐processing, including genotype filtering and annotation with snpeff v4.3t (Cingolani *et al.*, 2012) settled on approach that required > 10% base‐call supporting a SNP in the evolved genomes and < 2% base‐call supporting the same base in the parental genome data and genotype quality above 20. To detect chromosome copy number changes, we use the nquire tool (Weiß *et al.*, 2018) with mapping quality and minimum coverage filters set to 10 and applying lrdmodel to assess ploidy level.

### Statistical analysis

Sample averages were compared using Student's *t*‐test with the Excel software (Microsoft). *P* < 0.01 (**) and *P* < 0.05 (*,#) were considered statistically significant.

## Conflict of interest

The authors declare they have no conflict of interest.

## Supporting information


**Fig. S1.** NLs and PLs abundance.
**Fig. S2.** Knock‐out of some elongase genes depresses growth of yeast cells at high temperature.
**Fig. S3.** Histogram of cell count by DNA content of experimental populations.
**Fig. S4.** Ploidy‐specific growth effects under different stressful conditions.
**Table S1.** Relative abundance of Cer molecular species found in the parental yeast strain LH and their corresponding myriocin‐evolved clones LH09 and LH03.
**Table S2.** Relative abundance of IPC molecular species found in the parental yeast strain LH and their corresponding myriocin‐evolved clones LH09 and LH03.
**Table S3.** Relative abundance of MIPC molecular species found in the parental yeast strain LH and their corresponding myriocin‐evolved clones LH03 and LH09.
**Table S4.** Composition, chain length and degree of unsaturation of TAG molecular species found in the parental yeast strain LH and their corresponding myriocin‐evolved clones LH03 and LH09.
**Table S5.** Composition, chain length and degree of unsaturation of SE molecular species found in the parental yeast strain LH and their corresponding myriocin‐evolved clones LH03 and LH09.
**Table S6.** Composition, chain length and degree of unsaturation of DAG molecular species found in the parental yeast strain LH and their corresponding myriocin‐evolved clones LH03 and LH09.
**Table S7.** Composition, chain length and degree of unsaturation of PA molecular species found in the parental yeast strain LH and their corresponding myriocin‐evolved clones LH03 and LH09.
**Table S8.** Composition, chain length and degree of unsaturation of PC molecular species found in the parental yeast strain LH and their corresponding myriocin‐evolved clones LH03 and LH09.
**Table S9.** Composition, chain length and degree of unsaturation of PE molecular species found in the parental yeast strain LH and their corresponding myriocin‐evolved clones LH03 and LH09.
**Table S10.** Composition, chain length and degree of unsaturation of PG molecular species found in the parental yeast strain LH and their corresponding myriocin‐evolved clones LH03 and LH09.
**Table S11.** Composition, chain length and degree of unsaturation of PI molecular species found in the parental yeast strain LH and their corresponding myriocin‐evolved clones LH03 and LH09.
**Table S12.** Composition, chain length and degree of unsaturation of PS molecular species found in the parental yeast strain LH and their corresponding myriocin‐evolved clones LH03 and LH09.
**Table S13.** Single point differences between parental (LH) and evolved strain LH03^a^.
**Table S14.** Single point differences between parental (LH) and evolved strain LH09^a^.
**Table S15.** Single point differences with the parental strain (LH) common in both evolved strains (LH03 and LH09)^a^.
