# Supplementary material for: Myriocin‐induced adaptive laboratory evolution of an industrial strain of Saccharomyces cerevisiae reveals its potential to remodel lipid composition and heat tolerance
Source: Microb Biotechnol. 2020 Mar 25;13(4):1066–81. doi: 10.1111/1751-7915.13555 (PMC7264895; doi:10.1111/1751-7915.13555)
Supplement: Supplementary file 1 — Fig. S1. NLs and PLs abundance. Fig. S2. Knock‐out of some elongase genes depresses growth of yeast cells at high temperature. Fig. S3. Histogram of cell count by DNA content of experimental populations. Fig. S4. Ploidy‐specific growth effects under different stressful conditions. Table S1. Relative abundance of Cer molecular species found in the parental yeast strain LH and their corresponding myriocin‐evolved clones LH09 and LH03. Table S2. Relative abundance of IPC molecular species found in the parental yeast strain LH and their corresponding myriocin‐evolved clones LH09 and LH03. Table S3. Relative abundance of MIPC molecular species found in the parental yeast strain LH and their corresponding myriocin‐evolved clones LH03 and LH09. Table S4. Composition, chain length and degree of unsaturation of TAG molecular species found in the parental yeast strain LH and their corresponding myriocin‐evolved clones LH03 and LH09. Table S5. Composition, chain length and degree of unsaturation of SE molecular species found in the parental yeast strain LH and their corresponding myriocin‐evolved clones LH03 and LH09. Table S6. Composition, chain length and degree of unsaturation of DAG molecular species found in the parental yeast strain LH and their corresponding myriocin‐evolved clones LH03 and LH09. Table S7. Composition, chain length and degree of unsaturation of PA molecular species found in the parental yeast strain LH and their corresponding myriocin‐evolved clones LH03 and LH09. Table S8. Composition, chain length and degree of unsaturation of PC molecular species found in the parental yeast strain LH and their corresponding myriocin‐evolved clones LH03 and LH09. Table S9. Composition, chain length and degree of unsaturation of PE molecular species found in the parental yeast strain LH and their corresponding myriocin‐evolved clones LH03 and LH09. Table S10. Composition, chain length and degree of unsaturation of PG molecular species found in the parental yeast strain [file MBT2-13-1066-s001.docx]

**Supplementary information**

**Myriocin-induced adaptive laboratory evolution of an industrial strain of *Saccharomyces cerevisiae* reveals its potential to remodel lipid composition and heat tolerance**

**Francisca Randez-Gil, Jose A. Prieto, Alejandro Rodríguez-Puchades, Josefina Casas, Vicente Sentandreu**

**and Francisco Estruch**

**Contents:**

**Fig. S1-S4**

**Tables S1-S15**

**Fig. S1.** NLs and PLs abundance. Lipid profiles of the parental LH and the evolved clones LH03 and LH09 in YPD were analysed for neutral lipids (NLs) and phospholipids (PLs) classes. 30ºC-grown (OD_600_ ~ 1.0) cells were analysed by mass-spectrometry-based shotgun lipidomics. The amount of NLs, PLs, and their corresponding classes were normalized to the total lipid content and expressed as the mol%. Data represent the mean value (± SD) of three independent biological replicates. More details are given in the Experimental procedures section.

**Fig. S2**. Knock-out of some elongase genes depresses growth of yeast cells at high temperature. Cells of the laboratory wild-type BY4741 strain (wt) and its corresponding elongase mutants, *elo1*, *elo2* and *elo3*, were pre-grown in liquid YPD medium, refreshed in the same medium (initial OD_600_ ~ 0.05) and their growth was followed at 40ºC for the indicated time. Growth of the hydroxylase mutant *scs7* is also shown. Data represent the mean value (± SD) of three biological replicates.


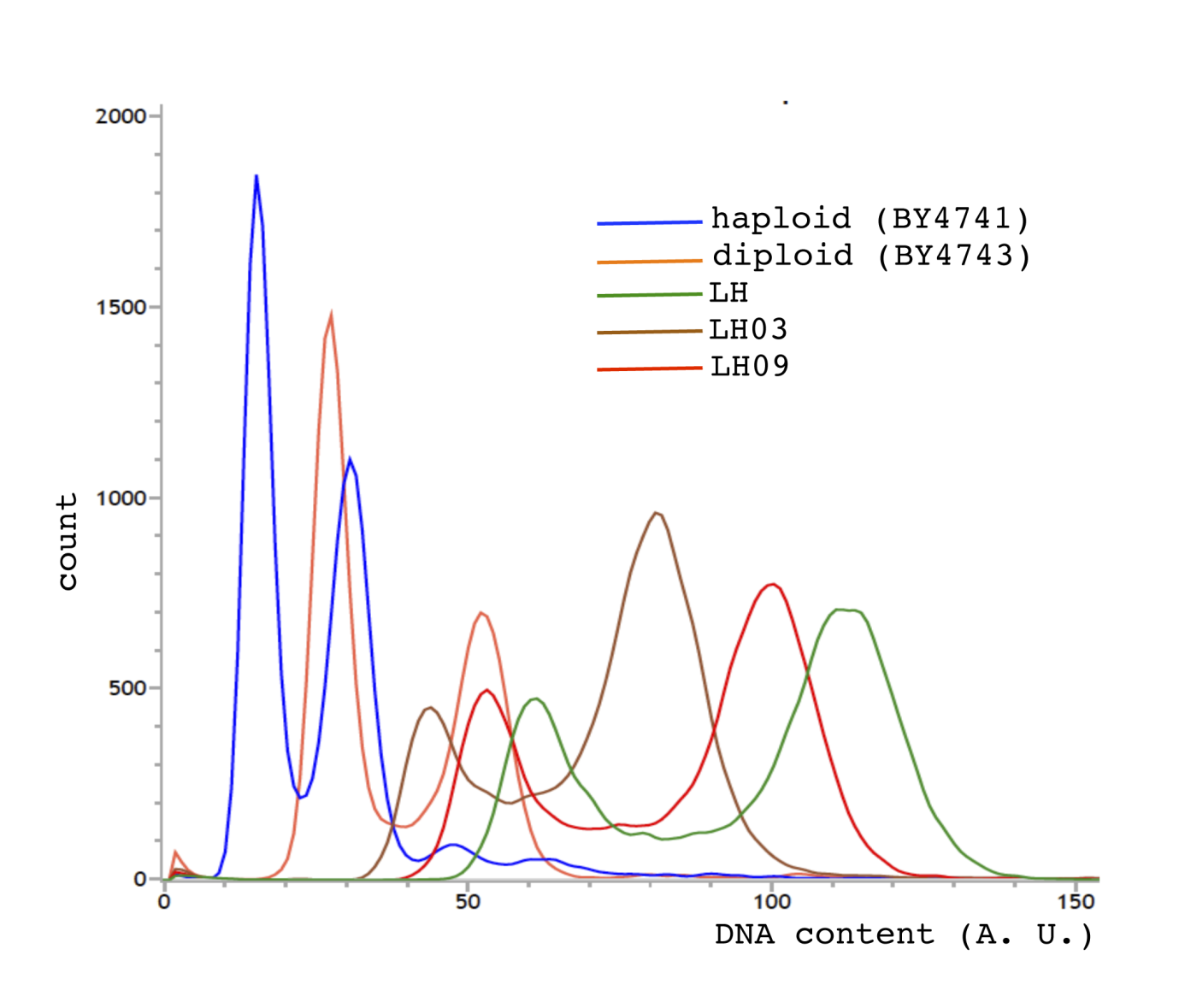


**Fig. S3.** Histogram of cell count by DNA content of experimental populations. The industrial LH strain and the evolved clones LH03 and LH09 were analysed for ploidy level by flow cytometry. Strains of known ploidy size, BY4741 (1N) and BY4743 (2N) were used as a genome size control. More details are given in the Experimental procedures section. A representative experiment is shown.

**Fig. S4.** Ploidy-specific growth effects under different stressful conditions. A serie of isogenic BY4741-derivative strains differing in ploidy level (1N, 2N, 3N and 4N; Storchová *et al*., 2006) were analysed for growth. Cells were grown in minimal SCD medium to the mid-exponential phase at 30ºC (OD_600_ ~ 0.5). Then, 10-fold serial dilutions were prepared and 3 μl aliquots of three dilutions (10–10^3^) were applied over SCD agar-gelled plates containing 2 μM aureobasidin A (AbA), 1.2 μM myriocin (Myr), 0.25 μg/ml soraphen A (SorA), 0.4 μM tunicamycin (Tn), 2 μM H_2_O_2_ (H_2_O_2_) or 1.0 M NaCl (NaCl). Colony growth was inspected after 2-4 days of incubation at 30ºC. In all cases, a representative experiment is shown.

**TABLE S1**. Relative abundance of Cer molecular species found in the parental yeast strain LH and their corresponding myriocin-evolved clones LH09 and LH03.

|  | | | | | | | | | | | |
| --- | --- | --- | --- | --- | --- | --- | --- | --- | --- | --- | --- |
| Cer specie |  | % of total Cer class ± SD^a^ | | | | | | | | | |
|  |  |  | | |  |  |  | |  | |  |
| DhC |  | LH |  | LH09 | | | |  | | LH03 | |
|  |  |  |  |  | | | |  | |  | |
| DhC (d18:0/20:0) |  | 0.03 ± 0.00 |  | 0.02 ± 0.00 | | | |  | | 0.04 ± 0.01^*#^ | |
| DhC (d18:0/22:0) |  | 0.06 ± 0.01 |  | 0.05 ± 0.01 | | | |  | | 0.06 ± 0.01 | |
| DhC (d18:0/24:0) |  | 0.24 ± 0.02 |  | 0.16 ± 0.02^*^ | | | |  | | 0.24 ± 0.03^#^ | |
|  |  |  |  |  | | | |  | |  | |
| PhC-B |  |  |  |  | | | |  | |  | |
| PhC (t32:0) |  | 0.01 ± 0.00 |  | nd | | | |  | | 0.02 ± 0.01^*^ | |
| PhC (t34:0) |  | 0.06 ± 0.00 |  | 0.05 ± 0.00^*^ | | | |  | | 0.10 ± 0.01^*#^ | |
| PhC (t36:0) |  | 0.10 ± 0.02 |  | 0.07 ± 0.01^*^ | | | |  | | 0.17 ± 0.01^*#^ | |
| PhC (t38:0) |  | 0.11 ± 0.01 |  | 0.07 ± 0.00^*^ | | | |  | | 0.16 ± 0.02^*#^ | |
| PhC (t40:0) |  | 0.12 ± 0.01 |  | 0.07 ± 0.01^*^ | | | |  | | 0.19 ± 0.06^#^ | |
| PhC (t42:0) |  | 1.33 ± 0.14 |  | 0.73 ± 0.11^*^ | | | |  | | 2.39 ± 0.58^*#^ | |
| PhC (t44:0) |  | 14.34 ± 1.65 |  | 14.62 ± 0.22 | | | |  | | 23.53 ± 0.99^*#^ | |
| PhC (t46:0) |  | 12.05 ± 1.56 |  | 11.14 ± 0.67 | | | |  | | 15.65 ± 1.65^*#^ | |
|  |  |  |  |  | | | |  | |  | |
| PhC-C |  |  |  |  | | | |  | |  | |
|  |  |  |  |  | | | |  | |  | |
| PhC (t34:0(2OH)) |  | 0.04 ± 0.01 |  | 0.04 ± 0.00 | | | |  | | 0.03 ± 0.00^*#^ | |
| PhC (t36:0(2OH)) |  | 0.07 ± 0.01 |  | 0.05 ± 0.01 | | | |  | | 0.04 ± 0.00^*#^ | |
| PhC (t38:0(2OH)) |  | 0.05 ± 0.02 |  | 0.05 ± 0.01 | | | |  | | 0.05 ± 0.02 | |
| PhC (t40:0(2OH)) |  | 0.11 ± 0.03 |  | 0.07 ± 0.00^*^ | | | |  | | 0.09 ± 0.02 | |
| PhC (t42:0(2OH)) |  | 4.40 ± 1.40 |  | 2.52 ± 0.35^*^ | | | |  | | 4.88 ± 1.53^#^ | |
| PhC (t44:0(2OH)) |  | 39.36 ± 3.30 |  | 40.70 ± 0.93 | | | |  | | 34.16 ± 2.33^*#^ | |
| PhC (t46:0(2OH)) |  | 27.53 ± 2.06 |  | 29.57 ± 0.39 | | | |  | | 18.21 ± 1.43^*#^ | |

^a^ Values are mean ± SD of three independent replicates. Significance level: ^*,#^ p < 0.05. ^*^ Significant difference of LH03 or LH09 compared with LH. ^#^ Significant difference of LH03 compared with LH09. nd, non detected.

**TABLE S2**. Relative abundance of IPC molecular species found in the parental yeast strain LH and their corresponding myriocin-evolved clones LH09 and LH03.

|  | | | | | | | |
| --- | --- | --- | --- | --- | --- | --- | --- |
| IPC specie |  | % of total IPC class ± SD^a^ | | | | |  |
|  |  |  |  |  |  |  |  |
| IPC-A |  | LH |  | LH09 |  | LH03 |  |
|  |  |  |  |  |  |  |  |
| IPC (d38:0) |  | nd |  | nd |  | nd |  |
| IPC (d40:0) |  | nd |  | nd |  | nd |  |
| IPC (d42:0) |  | nd |  | nd |  | nd |  |
| IPC (d44:0) |  | 0.62 ± 0.06 |  | 0.60 ± 0.05 |  | 0.88 ± 0.18^*#^ |  |
| IPC (d46:0) |  | nd |  | nd |  | nd |  |
|  |  |  |  |  |  |  |  |
| IPC-B |  |  |  |  |  |  |  |
| IPC (t38:0)/IPC (d38:0(2OH))  ((iunbcdndnjiiindvinds (d38:0(2OH)) (d38:0(2OH)) (d38:0(2OH)) |  | nd |  | nd |  | nd |  |
| IPC (t40:0)/IPC (d40:0(2OH))  ((iunbcdndnjiiindvinds (d38:0(2OH)) (d38:0(2OH)) (d38:0(2OH)) |  | nd |  | nd |  | nd |  |
| IPC (t42:0)/IPC (d42:0(2OH))  ((iunbcdndnjiiindvinds (d38:0(2OH)) (d38:0(2OH)) (d38:0(2OH)) |  | 0.41 ± 0.07 |  | 0.19 ± 0.03^*^ |  | 1.46 ± 0.55^*#^ |  |
| IPC (t44:0)/IPC (d44:0(2OH))  ((iunbcdndnjiiindvinds (d38:0(2OH)) (d38:0(2OH)) (d38:0(2OH)) |  | 10.73 ± 2.15 |  | 9.67 ± 1.85 |  | 24.32 ± 4.10^*#^ |  |
| IPC (t46:0)/IPC (d46:0(2OH))  ((iunbcdndnjiiindvinds (d38:0(2OH)) (d38:0(2OH)) (d38:0(2OH)) |  | nd |  | nd |  | nd |  |
|  |  |  |  |  |  |  |  |
| IPC-C |  |  |  |  |  |  |  |
|  |  |  |  |  |  |  |  |
| IPC (t38:0(2OH)) |  | 0.11 ± 0.04 |  | 0.06 ± 0.02 |  | 0.08 ± 0.04 |  |
| IPC (t40:0(2OH)) |  | nd |  | nd |  | nd |  |
| IPC (t42:0(2OH)) |  | 2.50 ± 0.08 |  | 1.29 ± 0.10^*^ |  | 3.52 ± 0.89^*^ |  |
| IPC (t44:0(2OH)) |  | 58.56 ± 2.79 |  | 58.33 ± 0.39 |  | 53.90 ± 2.14^*#^ |  |
| IPC (t46:0(2OH)) |  | 27.08 ± 1.27 |  | 29.86 ± 1.41 |  | 15.84 ± 3.76^*#^ |  |
|  |  |  |  |  |  |  |  |

^a^ Values are mean ± SD of three independent replicates. Significance level: ^*,#^ p< 0.05. ^*^ Significant difference of LH03 or LH09 compared with LH. ^#^ Significant difference of LH03 compared with LH09. nd, non detected.

**TABLE S3**. Relative abundance of MIPC molecular species found in the parental yeast strain LH and their corresponding myriocin-evolved clones LH03 and LH09.

|  | | | | | | | |
| --- | --- | --- | --- | --- | --- | --- | --- |
| MIPC specie |  | % of total MIPC class ± SD^a^ | | | | |  |
|  |  |  |  |  |  |  |  |
| MIPC-A |  | LH |  | LH09 |  | LH03 |  |
|  |  |  |  |  |  |  |  |
| MIPC (d38:0) |  | nd |  | nd |  | nd |  |
| MIPC (d40:0) |  | nd |  | nd |  | nd |  |
| MIPC (d42:0) |  | 0.22 ± 0.05 |  | nd |  | 0.41 ± 0.11^*^ |  |
| MIPC (d44:0) |  | 2.82 ± 0.53 |  | 2.61 ± 0.18 |  | 4.45 ± 0.63^*#^ |  |
| MIPC (d46:0) |  | 1.36 ± 0.32 |  | 1.24 ± 0.35 |  | 1.62 ± 0.79 |  |
|  |  |  |  |  |  |  |  |
| MIPC-B |  |  |  |  |  |  |  |
| MIPC (t38:0)/MIPC (d38:0(2OH)) ((iunbcdndnjiiindvinds (d38:0(2OH)) (d38:0(2OH)) (d38:0(2OH)) |  | nd |  | nd |  | nd |  |
| MIPC (t40:0)/MIPC (d40:0(2OH)) ((iunbcdndnjiiindvinds (d38:0(2OH)) (d38:0(2OH)) (d38:0(2OH)) |  | nd |  | nd |  | nd |  |
| MIPC (t42:0)/MIPC (d42:0(2OH)) ((iunbcdndnjiiindvinds (d38:0(2OH)) (d38:0(2OH)) (d38:0(2OH)) |  | 0.55 ± 0.18 |  | 0.40 ± 0.05 |  | 0.36 ± 0.12 |  |
| MIPC (t44:0)/MIPC (d44:0(2OH)) ((iunbcdndnjiiindvinds (d38:0(2OH)) (d38:0(2OH)) (d38:0(2OH)) |  | 8.11 ± 1.26 |  | 5.83 ± 0.58^*^ |  | 16.84 ± 2.47^*#^ |  |
| MIPC (t46:0)/MIPC (d46:0(2OH)) ((iunbcdndnjiiindvinds (d38:0(2OH)) (d38:0(2OH)) (d38:0(2OH)) |  | 19.76 ± 5.01 |  | 14.51 ± 1.85 |  | 27.44 ± 4.78^#^ |  |
|  |  |  |  |  |  |  |  |
| MIPC-C |  |  |  |  |  |  |  |
|  |  |  |  |  |  |  |  |
| MIPC (t38:0(2OH)) |  | nd |  | nd |  | nd |  |
| MIPC (t40:0(2OH)) |  | nd |  | nd |  | nd |  |
| MIPC (t42:0(2OH)) |  | 0.31 ± 0.02 |  | nd |  | 0.32 ± 0.07 |  |
| MIPC (t44:0(2OH)) |  | 48.53 ± 3.35 |  | 49.54 ± 2.92 |  | 53.02 ± 4.38 |  |
| MIPC (t46:0(2OH)) |  | 40.87 ± 3.33 |  | 42.37 ± 3.17 |  | 27.24 ± 5.58^*#^ |  |
|  |  |  |  |  |  |  |  |

^a^ Values are mean ± SD of three independent replicates. Significance level: ^*,#^ p < 0.05. ^*^ Significant difference of LH03 or LH09 compared with LH. ^#^ Significant difference of LH03 compared with LH09. nd, non detected.

**TABLE S4**. Composition, chain length and degree of unsaturation of TAG molecular species found in the parental yeast strain LH and their corresponding myriocin-evolved clones LH03 and LH09.

|  | | | | | | | | | | | | | |
| --- | --- | --- | --- | --- | --- | --- | --- | --- | --- | --- | --- | --- | --- |
|  |  | mol % of total TAG ± SD^a^ | | | | | | | | | | | |
|  |  |  | |  | |  |  |  | | | | |  |
| TAG specie |  | LH |  | | LH09 | | | |  | | LH03 | | |
|  |  |  |  | |  | | | | |  | |  | |
| C42:1 |  | 2.25 ± 1.59 |  | | 4.19 ± 0.38 | | | | |  | | 4.85 ± 0.19^#^ | |
| C42:2 |  | 3.14 ± 0.46 |  | | 3.87 ± 0.13^*^ | | | | |  | | 4.12 ± 2.38 | |
| C44:2 |  | 4.07 ± 0.49 |  | | 4.45 ± 0.22 | | | | |  | | 4.84 ± 0.23^*^ | |
| C48:3 |  | 13.23 ± 0.83 |  | | 16.00 ± 0.71^*^ | | | | |  | | 17.92 ± 1.01^*#^ | |
| C50:3 |  | 42.89 ± 1.64 |  | | 41.63 ± 1.22 | | | | |  | | 41.42 ± 0.48 | |
| C52:3 |  | 32.06 ± 2.83 |  | | 24.31 ± 1.11^*^ | | | | |  | | 22.37 ± 1.51^*^ | |
| C56:1 |  | nd |  | | 2.56 ± 1.57 | | | | |  | | 1.37 ± 0.79 | |
| C56:2 |  | 2.39 ± 1.69 |  | | 2.48 ± 1.44 | | | | |  | | 2.12 ± 1.23 | |
| C56:3 |  | 1.70 ± 0.16 |  | | 1.58 ± 0.93 | | | | |  | | 1.53 ± 0.88 | |
| C58:1 |  | nd |  | | 1.49 ± 0.86 | | | | |  | | 1.17 ± 0.68 | |
| C58:2 |  | 1.18 ± 0.84 |  | | 1.92 ± 1.11 | | | | |  | | 2.28 ± 1.34 | |
| C60:2 |  | nd |  | | nd | | | | |  | | 3.41 ± 1.97 | |
|  |  |  |  | |  | | | | |  | |  | |
| Total chain length |  |  |  | |  | | | | |  | |  | |
|  |  |  |  | |  | | | | |  | |  | |
| C42 |  | 4.27 ± 1.13 |  | | 8.06 ± 0.45^*^ | | | | |  | | 8.86 ± 0.20^*^ | |
| C44 |  | 4.07 ± 0.49 |  | | 4.45 ± 0.22 | | | | |  | | 4.84 ± 0.23^*^ | |
| C48 |  | 13.23 ± 0.83 |  | | 16.00 ± 0.71^*^ | | | | |  | | 17.92 ± 1.01^*#^ | |
| C50 |  | 42.89 ± 1.64 |  | | 41.63 ± 1.22 | | | | |  | | 41.42 ± 0.48 | |
| C52 |  | 32.06 ± 2.83 |  | | 24.31 ± 1.11^*^ | | | | |  | | 22.37 ± 1.51^*^ | |
| C56 |  | 2.89 ± 1.85 |  | | 6.63 ± 0.42 | | | | |  | | 2.51 ± 1.62^#^ | |
| C58 |  | 1.18 ± 0.68 |  | | 3.41 ± 1.97 | | | | |  | | 2.87 ± 1.19 | |
| C60 |  | nd |  | | nd | | | | |  | | 3.41 ± 0.14 | |
|  |  |  |  | |  | | | | |  | |  | |
| Degree of unsaturation |  |  |  | |  | | | | |  | |  | |
|  |  |  |  | |  | | | | |  | |  | |
| C:1 |  | 3.17 ± 2.05 |  | | 6.39 ± 1.04^*^ | | | | |  | | 5.70 ± 0.56^*^ | |
| C:2 |  | 9.33 ± 1.40 |  | | 10.62 ± 0.38 | | | | |  | | 12.08 ± 0.62^*#^ | |
| C:3 |  | 88.55 ± 2.31 |  | | 82.99 ± 1.38^*^ | | | | |  | | 82.22 ± 0.63^*^ | |

^a^ Values are mean ± SD of three independent replicates. Significance level: ^*,#^ p < 0.05. ^*^ Significant difference of LH03 or LH09 compared with LH. ^#^ Significant difference of LH03 compared with LH09. nd, non detected.

**TABLE S5**. Composition, chain length and degree of unsaturation of SE molecular species found in the parental yeast strain LH and their corresponding myriocin-evolved clones LH03 and LH09.

|  | | | | | | | | | | | | | |
| --- | --- | --- | --- | --- | --- | --- | --- | --- | --- | --- | --- | --- | --- |
|  |  | mol % of total SE ± SD^a^ | | | | | | | | | | | |
|  |  |  | |  | |  |  |  | | | | |  |
| SE specie |  | LH |  | | LH09 | | | |  | | LH03 | | |
|  |  |  |  | |  | | | | |  | |  | |
| C16:0  EE 16:1  EE 18:1 |  | 25.33 ± 2.13 |  | | 26.55 ± 2.51 | | | | |  | | 26.06 ± 2.22 | |
| C16:1 |  | 40.44 ± 3.03 |  | | 41.26 ± 1.47 | | | | |  | | 46.09 ± 2.18 | |
| C18:0 |  | 34.23 ± 0.90 |  | | 32.19 ± 3.98 | | | | |  | | 27.85 ± 0.04^*^ | |
|  |  |  |  | |  | | | | |  | |  | |
| Total chain length |  |  |  | |  | | | | |  | |  | |
|  |  |  |  | |  | | | | |  | |  | |
| C16 |  | 65.77 ± 0.90 |  | | 67.81 ± 3.98 | | | | |  | | 72.15 ± 0.04 | |
| C18 |  | 34.23 ± 0.90 |  | | 32.19 ± 3.98 | | | | |  | | 27.85 ± 0.04^*^ | |
|  |  |  |  | |  | | | | |  | |  | |
| Degree of unsaturation |  |  |  | |  | | | | |  | |  | |
|  |  |  |  | |  | | | | |  | |  | |
| C:0 |  | 25.33 ± 2.13 |  | | 26.55 ± 2.51 | | | | |  | | 26.06 ± 2.22 | |
| C:1 |  | 74.67 ± 2.13 |  | | 73.45 ± 2.51 | | | | |  | | 73.94 ± 2.22 | |

^a^ Values are mean ± SD of three independent replicates. Significance level: ^*,#^ p < 0.05. ^*^ Significant difference of LH03 or LH09 compared with LH. ^#^ Significant difference of LH03 compared with LH09.

**TABLE S6**. Composition, chain length and degree of unsaturation of DAG molecular species found in the parental yeast strain LH and their corresponding myriocin-evolved clones LH03 and LH09.

|  | | | | | | | | | | | | | |
| --- | --- | --- | --- | --- | --- | --- | --- | --- | --- | --- | --- | --- | --- |
|  |  | mol % of total DAG ± SD^a^ | | | | | | | | | | | |
|  |  |  | |  | |  |  |  | | | | |  |
| DAG specie |  | LH |  | | LH09 | | | |  | | LH03 | | |
|  |  |  |  | |  | | | | |  | |  | |
| C30:2 |  | 0.72 ± 0.30 |  | | 0.43 ± 0.06 | | | | |  | | 0.51 ± 0.08 | |
| C32:1 |  | 12.76 ± 0.43 |  | | 17.36 ± 0.67^*^ | | | | |  | | 19.25 ± 0.29^*#^ | |
| C32:2 |  | 16.35 ± 0.00 |  | | 19.62 ± 0.18^*^ | | | | |  | | 19.10 ± 0.52^*^ | |
| C34:1 |  | 31.71 ± 1.82 |  | | 32.52 ± 0.57 | | | | |  | | 33.41 ± 0.42^#^ | |
| C34:2 |  | 32.64 ± 3.34 |  | | 30.07 ± 1.28 | | | | |  | | 27.73 ± 0.76^*#^ | |
| C36:2 |  | 5.82 ± 0.79 |  | | nd | | | | |  | |  | |
|  |  |  |  | |  | | | | |  | |  | |
| Total chain length |  |  |  | |  | | | | |  | |  | |
|  |  |  |  | |  | | | | |  | |  | |
| C30 |  | 0.72 ± 0.30 |  | | 0.43 ± 0.06 | | | | |  | | 0.51 ± 0.08 | |
| C32 |  | 29.11 ± 0.42 |  | | 36.98 ± 0.78^*^ | | | | |  | | 38.35 ± 0.80^*^ | |
| C34 |  | 64.35 ± 1.52 |  | | 62.59 ± 0.83 | | | | |  | | 61.14 ± 0.86^*^ | |
| C36 |  | 5.82 ± 0.79 |  | | nd | | | | |  | |  | |
|  |  |  |  | |  | | | | |  | |  | |
| Degree of unsaturation |  |  |  | |  | | | | |  | |  | |
|  |  |  |  | |  | | | | |  | |  | |
| C:1 |  | 44.47 ± 2.25 |  | | 49.88 ± 1.16^*^ | | | | |  | | 52.66 ± 0.37^*#^ | |
| C:2 |  | 55.53 ± 2.25 |  | | 50.12 ± 1.16^*^ | | | | |  | | 47.34 ± 0.37^*#^ | |

^a^ Values are mean ± SD of three independent replicates. Significance level: ^*,#^ p < 0.05. ^*^ Significant difference of LH03 or LH09 compared with LH. ^#^ Significant difference of LH03 compared with LH09. nd, non detected.

**TABLE S7**. Composition, chain length and degree of unsaturation of PA molecular species found in the parental yeast strain LH and their corresponding myriocin-evolved clones LH03 and LH09.

|  | | | | | | | | | | | | | |
| --- | --- | --- | --- | --- | --- | --- | --- | --- | --- | --- | --- | --- | --- |
|  |  | mol % of total PA ± SD^a^ | | | | | | | | | | | |
|  |  |  | |  | |  |  |  | | | | |  |
| PA specie |  | LH |  | | LH09 | | | |  | | LH03 | | |
|  |  |  |  | |  | | | | |  | |  | |
| C28:1  PA 30:1  PA 30:2  PA 32:1  PA 32:2  PA 34:1  PA 34:2  PA 36:2 |  | 1.82 ± 0.27 |  | | nd | | | | |  | | 1.85 ± 0.21 | |
| C30:1 |  | 1.92 ± 0.17 |  | | 2.11 ± 0.09 | | | | |  | | 2.54 ± 0.12^*#^ | |
| C30:2 |  | 1.58 ± 0.01 |  | | nd | | | | |  | | 1.15 ± 0.07^*^ | |
| C32:1 |  | 7.89 ± 0.56 |  | | 11.77 ± 0.61^*^ | | | | |  | | 12.53 ± 0.57^*^ | |
| C32:2 |  | 27.49 ± 1.07 |  | | 27.10 ± 0.50 | | | | |  | | 27.52 ± 1.30 | |
| C34:1 |  | 15.30 ± 0.20 |  | | 19.08 ± 1.15^*^ | | | | |  | | 21.01 ± 1.75^*^ | |
| C34:2 |  | 41.67 ± 0.89 |  | | 37.47 ± 1.65^*^ | | | | |  | | 32.32 ± 0.26^*#^ | |
| C36:2 |  | 3.47 ± 0.29 |  | | 2.46 ± 0.55^*^ | | | | |  | | 1.47 ± 0.04^*#^ | |
|  |  |  |  | |  | | | | |  | |  | |
| Total chain length |  |  |  | |  | | | | |  | |  | |
|  |  |  |  | |  | | | | |  | |  | |
| C28 |  | 1.82 ± 0.27 |  | | nd | | | | |  | | 1.85 ± 0.21 | |
| C30 |  | 2.97 ± 0.74 |  | | 2.11 ± 0.09 | | | | |  | | 3.31 ± 0.76 | |
| C32 |  | 35.37 ± 1.43 |  | | 38.88 ± 1.01^*^ | | | | |  | | 40.05 ± 1.20^*^ | |
| C34 |  | 56.97 ± 1.07 |  | | 56.55 ± 0.52 | | | | |  | | 53.32 ± 1.50^*#^ | |
| C36 |  | 3.47 ± 0.29 |  | | 2.46 ± 0.55^*^ | | | | |  | | 1.47 ± 0.04^*#^ | |
|  |  |  |  | |  | | | | |  | |  | |
| Degree of unsaturation |  |  |  | |  | | | | |  | |  | |
|  |  |  |  | |  | | | | |  | |  | |
| C:1 |  | 26.31 ± 0.79 |  | | 32.97 ± 1.83^*^ | | | | |  | | 37.93 ± 1.97^*#^ | |
| C:2 |  | 73.69 ± 0.79 |  | | 67.03 ± 1.83^*^ | | | | |  | | 62.07 ± 1.97^*#^ | |
|  |  |  |  | |  | | | | |  | |  | |

^a^ Values are mean ± SD of three independent replicates. Significance level: ^*,#^ p < 0.05. ^*^ Significant difference of LH03 or LH09 compared with LH. ^#^ Significant difference of LH03 compared with LH09. nd, non detected.

**TABLE S8**. Composition, chain length and degree of unsaturation of PC molecular species found in the parental yeast strain LH and their corresponding myriocin-evolved clones LH03 and LH09.

|  | | | | | | | | | | | | | |
| --- | --- | --- | --- | --- | --- | --- | --- | --- | --- | --- | --- | --- | --- |
|  |  | mol % of total PC ± SD^a^ | | | | | | | | | | | |
|  |  |  | |  | |  |  |  | | | | |  |
| PC specie |  | LH |  | | LH09 | | | |  | | LH03 | | |
|  |  |  |  | |  | | | | |  | |  | |
| C26:0 |  | 0.28 ± 0.07 |  | | 0.47 ± 0.01^*^ | | | | |  | | 0.47 ± 0.04^*^ | |
| C26:1 |  | 2.79 ± 1.61 |  | | 3.81 ± 2.20^*^ | | | | |  | | 3.34 ± 0.06^*#^ | |
| C26:2 |  | 0.09 ± 0.01 |  | | 0.09 ± 0.00 | | | | |  | | 0.06 ± 0.00^*#^ | |
| C28:0 |  | 0.09 ± 0.06 |  | | 0.21 ± 0.00^*^ | | | | |  | | 0.24 ± 0.02^*#^ | |
| C28:1 |  | 2.48 ± 0.08 |  | | 2.90 ± 0.07^*^ | | | | |  | | 2.98 ± 0.10^*^ | |
| C28:2 |  | 0.08 ± 0.04 |  | | 0.07 ± 0.01 | | | | |  | | 0.06 ± 0.01^*#^ | |
| C30:0 |  | nd |  | | 0.05 ± 0.01 | | | | |  | | 0.08 ± 0.01^#^ | |
| C30:1 |  | 1.68 ± 0.17 |  | | 2.37 ± 0.08^*^ | | | | |  | | 3.05 ± 0.05^*#^ | |
| C30:2 |  | 1.60 ± 0.17 |  | | 1.34 ± 0.07^*^ | | | | |  | | 1.45 ± 0.09 | |
| C32:1 |  | 2.73 ± 0.56 |  | | 5.49 ± 0.29^*^ | | | | |  | | 6.20 ± 0.43^*#^ | |
| C32:2 |  | 36.98 ± 1.45 |  | | 38.59 ± 0.45 | | | | |  | | 43.03 ± 0.59^*#^ | |
| C34:1 |  | 1.18 ± 0.21 |  | | 2.30 ± 0.09^*^ | | | | |  | | 2.55 ± 0.20^*^ | |
| C34:2 |  | 44.53 ± 1.40 |  | | 38.73 ± 2.14^*^ | | | | |  | | 33.29 ± 0.31^*#^ | |
| C36:1 |  | 0.56 ± 0.12 |  | | 0.63 ± 0.03 | | | | |  | | 0.68 ± 0.04 | |
| C36:2 |  | 5.77 ± 0.40 |  | | 4.12 ± 0.54^*^ | | | | |  | | 2.47 ± 0.05^*#^ | |
| C38:2 |  | 0.15 ± 0.01 |  | | 0.09 ± 0.01^*^ | | | | |  | | 0.05 ± 0.00^*#^ | |
|  |  |  |  | |  | | | | |  | |  | |
| Total chain length |  |  |  | |  | | | | |  | |  | |
|  |  |  |  | |  | | | | |  | |  | |
| C26 |  | 3.20 ± 1.85 |  | | 4.36 ± 2.52^*^ | | | | |  | | 3.87 ± 0.03^*#^ | |
| C28 |  | 2.60 ± 0.13 |  | | 3.18 ± 0.08^*^ | | | | |  | | 3.28 ± 0.11^*^ | |
| C30 |  | 3.28 ± 0.09 |  | | 3.76 ± 0.08^*^ | | | | |  | | 4.58 ± 0.12^*#^ | |
| C32 |  | 39.71 ± 1.16 |  | | 44.09 ± 0.72^*^ | | | | |  | | 49.23 ± 0.32^*#^ | |
| C34 |  | 45.71 ± 1.19 |  | | 41.03 ± 2.13^*^ | | | | |  | | 35.84 ± 0.24^*#^ | |
| C36 |  | 6.33 ± 0.43 |  | | 4.75 ± 0.57^*^ | | | | |  | | 3.14 ± 0.04^*#^ | |
| C38 |  | 0.16 ± 0.02 |  | | 0.26 ± 0.02^*^ | | | | |  | | 0.33 ± 0.02^*#^ | |
|  |  |  |  | |  | | | | |  | |  | |
| Degree of unsaturation |  |  |  | |  | | | | |  | |  | |
|  |  |  |  | |  | | | | |  | |  | |
| C:0 |  | 0.34 ± 0.12 |  | | 0.73 ± 0.01^*^ | | | | |  | | 0.79 ± 0.05^*^ | |
| C:1 |  | 10.49 ± 2.61 |  | | 16.24 ± 2.34^*^ | | | | |  | | 18.80 ± 0.70^*^ | |
| C:2 |  | 89.17 ± 2.73 |  | | 83.03 ± 2.33^*^ | | | | |  | | 80.41 ± 0.75^*^ | |
|  |  |  |  | |  | | | | |  | |  | |

^a^ Values are mean ± SD of three independent replicates. Significance level: ^*,#^ p < 0.05. ^*^ Significant difference of LH03 or LH09 compared with LH. ^#^ Significant difference of LH03 compared with LH09. nd, non detected.

**TABLE S9**. Composition, chain length and degree of unsaturation of PE molecular species found in the parental yeast strain LH and their corresponding myriocin-evolved clones LH03 and LH09.

|  | | | | | | | | | | | | | |
| --- | --- | --- | --- | --- | --- | --- | --- | --- | --- | --- | --- | --- | --- |
|  |  | mol % of total PE ± SD^a^ | | | | | | | | | | | |
|  |  |  | |  | |  |  |  | | | | |  |
| PE specie |  | LH |  | | LH09 | | | |  | | LH03 | | |
|  |  |  |  | |  | | | | |  | |  | |
| C26:0 |  | 0.16 ± 0.09 |  | | 0.29 ± 0.17 | | | | |  | | 0.27 ± 0.03 | |
| C26:1 |  | 0.18 ± 0.10 |  | | 0.27 ± 0.15 | | | | |  | | 0.19 ± 0.03 | |
| C28:0 |  | nd |  | | 0.16 ± 0.09 | | | | |  | | 0.16 ± 0.09 | |
| C28:1 |  | 0.31 ± 0.18 |  | | 0.36 ± 0.05 | | | | |  | | 0.33 ± 0.03 | |
| C30:1 |  | 0.67 ± 0.09 |  | | 0.79 ± 0.01^*^ | | | | |  | | 0.91 ± 0.08^*#^ | |
| C32:1 |  | 6.91 ± 0.37 |  | | 9.61 ± 0.58^*^ | | | | |  | | 11.73 ± 0.08^*#^ | |
| C32:2 |  | 11.24 ± 0.38 |  | | 14.12 ± 0.66^*^ | | | | |  | | 14.49 ± 0.16^*^ | |
| C34:1 |  | 14.38 ± 1.21 |  | | 16.02 ± 0.38^*^ | | | | |  | | 17.74 ± 0.45^*#^ | |
| C34:2 |  | 58.36 ± 0.89 |  | | 51.82 ± 1.00^*^ | | | | |  | | 50.02 ± 0.26^*#^ | |
| C36:2 |  | 8.12 ± 0.59 |  | | 7.05 ± 0.28^*^ | | | | |  | | 4.21 ± 0.10^*#^ | |
|  |  |  |  | |  | | | | |  | |  | |
| Total chain length |  |  |  | |  | | | | |  | |  | |
|  |  |  |  | |  | | | | |  | |  | |
| C26 |  | 0.17 ± 0.10 |  | | 0.55 ± 0.32 | | | | |  | | 0.46 ± 0.02 | |
| C28 |  | 0.31 ± 0.18 |  | | 0.42 ± 0.13 | | | | |  | | 0.44 ± 0.10 | |
| C30 |  | 0.67 ± 0.09 |  | | 0.79 ± 0.01^*^ | | | | |  | | 0.91 ± 0.08^*#^ | |
| C32 |  | 18.15 ± 0.24 |  | | 23.73 ± 1.22^*^ | | | | |  | | 26.22 ± 0.18^*#^ | |
| C34 |  | 72.74 ± 0.49 |  | | 67.83 ± 1.24^*^ | | | | |  | | 67.77 ± 0.21^*^ | |
| C36 |  | 8.12 ± 0.59 |  | | 7.05 ± 0.28^*^ | | | | |  | | 4.21 ± 0.10^*#^ | |
|  |  |  |  | |  | | | | |  | |  | |
| Degree of unsaturation |  |  |  | |  | | | | |  | |  | |
|  |  |  |  | |  | | | | |  | |  | |
| C:0 |  | 0.16 ± 0.09 |  | | 0.22 ± 0.09 | | | | |  | | 0.38 ± 0.11 | |
| C:1 |  | 22.22 ± 1.71 |  | | 26.87 ± 1.71^*^ | | | | |  | | 30.90 ± 0.33^*#^ | |
| C:2 |  | 77.73 ± 1.70 |  | | 72.98 ± 1.70^*^ | | | | |  | | 68.72 ± 0.24^*#^ | |
|  |  |  |  | |  | | | | |  | |  | |

^a^ Values are mean ± SD of three independent replicates. Significance level: ^*,#^ p < 0.05. ^*^ Significant difference of LH03 or LH09 compared with LH. ^#^ Significant difference of LH03 compared with LH09. nd, non detected.

**TABLE S10**. Composition, chain length and degree of unsaturation of PG molecular species found in the parental yeast strain LH and their corresponding myriocin-evolved clones LH03 and LH09.

|  | | | | | | | | | | | | | |
| --- | --- | --- | --- | --- | --- | --- | --- | --- | --- | --- | --- | --- | --- |
|  |  | mol % of total PG ± SD^a^ | | | | | | | | | | | |
|  |  |  | |  | |  |  |  | | | | |  |
| PG specie |  | LH |  | | LH09 | | | |  | | LH03 | | |
|  |  |  |  | |  | | | | |  | |  | |
| C32:1 |  | 19.85 ± 0.24 |  | | 25.85 ± 2.17^*^ | | | | |  | | 27.85 ± 0.51^*^ | |
| C32:2 |  | 8.69 ± 0.88 |  | | 7.31 ± 0.40^*^ | | | | |  | | 7.46 ± 1.40 | |
| C34:1 |  | 49.42 ± 3.95 |  | | 51.17 ± 2.40 | | | | |  | | 50.39 ± 0.95 | |
| C34:2 |  | 22.04 ± 2.84 |  | | 15.67 ± 0.43^*^ | | | | |  | | 14.30 ± 0.44^*#^ | |
|  |  |  |  | |  | | | | |  | |  | |
| Total chain length |  |  |  | |  | | | | |  | |  | |
|  |  |  |  | |  | | | | |  | |  | |
| C32 |  | 28.54 ± 1.11 |  | | 33.16 ± 2.00^*^ | | | | |  | | 35.31 ± 1.02^*^ | |
| C34 |  | 71.46 ± 1.11 |  | | 66.84 ± 2.00^*^ | | | | |  | | 64.69 ± 1.02^*^ | |
|  |  |  |  | |  | | | | |  | |  | |
| Degree of unsaturation |  |  |  | |  | | | | |  | |  | |
|  |  |  |  | |  | | | | |  | |  | |
| C:1 |  | 69.26 ± 3.71 |  | | 77.02 ± 0.33^*^ | | | | |  | | 78.25 ± 1.43^*^ | |
| C:2 |  | 30.74 ± 3.71 |  | | 22.98 ± 0.33^*^ | | | | |  | | 21.75 ± 1.43^*^ | |
|  |  |  |  | |  | | | | |  | |  | |

^a^ Values are mean ± SD of three independent replicates. Significance level: ^*,#^ p < 0.05. ^*^ Significant difference of LH03 or LH09 compared with LH. ^#^ Significant difference of LH03 compared with LH09. nd, non detected.

**TABLE S11**. Composition, chain length and degree of unsaturation of PI molecular species found in the parental yeast strain LH and their corresponding myriocin-evolved clones LH03 and LH09.

|  | | | | | | | | | | | | | |
| --- | --- | --- | --- | --- | --- | --- | --- | --- | --- | --- | --- | --- | --- |
|  |  | mol % of total PI ± SD^a^ | | | | | | | | | | | |
|  |  |  | |  | |  |  |  | | | | |  |
| PI specie |  | LH |  | | LH09 | | | |  | | LH03 | | |
|  |  |  |  | |  | | | | |  | |  | |
| C26:0 |  | 2.27 ± 0.24 |  | | 3.73 ± 0.42^*^ | | | | |  | | 3.60 ± 0.16^*^ | |
| C26:1 |  | 0.98 ± 0.57 |  | | 1.37 ± 0.80 | | | | |  | | 1.06 ± 0.62 | |
| C28:0 |  | 1.74 ± 0.11 |  | | 2.33 ± 0.17^*^ | | | | |  | | 2.34 ± 0.11^*^ | |
| C28:1 |  | 0.80 ± 0.08 |  | | 0.81 ± 0.04 | | | | |  | | 0.71 ± 0.09 | |
| C30:0 |  | 0.36 ± 0.01 |  | | 0.42 ± 0.02^*^ | | | | |  | | 0.53 ± 0.01^*#^ | |
| C30:1 |  | 1.56 ± 0.02 |  | | 1.58 ± 0.06 | | | | |  | | 1.77 ± 0.24 | |
| C30:2 |  | 0.24 ± 0.02 |  | | 0.18 ± 0.01^*^ | | | | |  | | 0.18 ± 0.04^*^ | |
| C32:1 |  | 16.74 ± 0.64 |  | | 22.07 ± 0.95^*^ | | | | |  | | 23.67 ± 0.70^*#^ | |
| C32:2 |  | 5.31 ± 0.24 |  | | 5.30 ± 0.32 | | | | |  | | 5.14 ± 0.63 | |
| C34:1 |  | 43.12 ± 1.54 |  | | 43.36 ± 1.41 | | | | |  | | 44.27 ± 1.85 | |
| C34:2 |  | 16.78 ± 1.34 |  | | 11.99 ± 0.60^*^ | | | | |  | | 10.81 ± 0.66^*#^ | |
| C36:1 |  | 8.62 ± 0.69 |  | | 6.12 ± 0.57^*^ | | | | |  | | 5.43 ± 0.44^*^ | |
| C36:2 |  | 2.15 ± 0.19 |  | | 1.20 ± 0.17^*^ | | | | |  | | 0.85 ± 0.03^*#^ | |
|  |  |  |  | |  | | | | |  | |  | |
| Total chain length |  |  |  | |  | | | | |  | |  | |
|  |  |  |  | |  | | | | |  | |  | |
| C26 |  | 2.59 ± 0.54 |  | | 4.65 ± 1.18^*^ | | | | |  | | 4.30 ± 0.47^*^ | |
| C28 |  | 2.54 ± 0.15 |  | | 3.14 ± 0.21^*^ | | | | |  | | 3.05 ± 0.18^*^ | |
| C30 |  | 2.16 ± 0.02 |  | | 2.17 ± 0.05 | | | | |  | | 2.48 ± 0.27 | |
| C32 |  | 22.04 ± 0.41 |  | | 27.36 ± 1.26^*^ | | | | |  | | 28.81 ± 1.33^*^ | |
| C34 |  | 59.89 ± 0.21 |  | | 55.35 ± 1.91^*^ | | | | |  | | 55.08 ± 1.22^*^ | |
| C36 |  | 10.77 ± 0.86 |  | | 7.32 ± 0.73^*^ | | | | |  | | 6.28 ± 0.41^*#^ | |
|  |  |  |  | |  | | | | |  | |  | |
| Degree of unsaturation |  |  |  | |  | | | | |  | |  | |
|  |  |  |  | |  | | | | |  | |  | |
| C:0 |  | 4.37 ± 0.35 |  | | 6.48 ± 0.58^*^ | | | | |  | | 6.47 ± 0.26^*^ | |
| C:1 |  | 71.16 ± 1.43 |  | | 74.85 ± 0.63^*^ | | | | |  | | 76.56 ± 1.51^*^ | |
| C:2 |  | 24.46 ± 1.78 |  | | 18.66 ± 0.52^*^ | | | | |  | | 16.98 ± 1.35^*^ | |
|  |  |  |  | |  | | | | |  | |  | |

^a^ Values are mean ± SD of three independent replicates. Significance level: ^*,#^ p < 0.05. ^*^ Significant difference of LH03 or LH09 compared with LH. ^#^ Significant difference of LH03 compared with LH09. nd, non detected.

**TABLE S12**. Composition, chain length and degree of unsaturation of PS molecular species found in the parental yeast strain LH and their corresponding myriocin-evolved clones LH03 and LH09.

|  | | | | | | | | | | | | | |
| --- | --- | --- | --- | --- | --- | --- | --- | --- | --- | --- | --- | --- | --- |
|  |  | mol % of total PS ± SD^a^ | | | | | | | | | | | |
|  |  |  | |  | |  |  |  | | | | |  |
| PS specie |  | LH |  | | LH09 | | | |  | | LH03 | | |
|  |  |  |  | |  | | | | |  | |  | |
| C30:1 |  | 0.80 ± 0.46 |  | | 0.70 ± 0.15 | | | | |  | | 0.85 ± 0.18 | |
| C32:1 |  | 12.52 ± 0.62 |  | | 16.66 ± 0.63^*^ | | | | |  | | 17.57 ± 0.70^*^ | |
| C32:2 |  | 5.32 ± 0.20 |  | | 5.68 ± 0.26 | | | | |  | | 5.75 ± 0.12^*^ | |
| C34:1 |  | 42.36 ± 1.14 |  | | 45.71 ± 0.72^*^ | | | | |  | | 47.63 ± 0.91^*#^ | |
| C34:2 |  | 36.20 ± 1.63 |  | | 28.96 ± 0.73^*^ | | | | |  | | 25.92 ± 0.23^*#^ | |
| C36:1 |  | 1.24 ± 0.08 |  | | 1.20 ± 0.13^*^ | | | | |  | | 1.36 ± 0.02^*^ | |
| C36:2 |  | 1.83 ± 0.12 |  | | 1.08 ± 0.03^*^ | | | | |  | | 0.91 ± 0.06^*#^ | |
|  |  |  |  | |  | | | | |  | |  | |
| Total chain length |  |  |  | |  | | | | |  | |  | |
|  |  |  |  | |  | | | | |  | |  | |
| C30 |  | 0.80 ± 0.46 |  | | 0.70 ± 0.15 | | | | |  | | 0.85 ± 0.18 | |
| C32 |  | 17.84 ± 0.42 |  | | 22.35 ± 0.87^*^ | | | | |  | | 23.31 ± 0.81^*^ | |
| C34 |  | 78.56 ± 0.61 |  | | 74.67 ± 0.97^*^ | | | | |  | | 73.55 ± 0.98^*^ | |
| C36 |  | 3.07 ± 0.19 |  | | 2.29 ± 0.12^*^ | | | | |  | | 2.28 ± 0.07^*^ | |
|  |  |  |  | |  | | | | |  | |  | |
| Degree of unsaturation |  |  |  | |  | | | | |  | |  | |
|  |  |  |  | |  | | | | |  | |  | |
| C:1 |  | 56.65 ± 1.94 |  | | 64.27 ± 0.71^*^ | | | | |  | | 67.42 ± 0.13^*#^ | |
| C:2 |  | 43.35 ± 1.94 |  | | 35.73 ± 0.71^*^ | | | | |  | | 32.58 ± 0.13^*#^ | |
|  |  |  |  | |  | | | | |  | |  | |

^a^ Values are mean ± SD of three independent replicates. Significance level: ^*,#^ p < 0.05. ^*^ Significant difference of LH03 or LH09 compared with LH. ^#^ Significant difference of LH03 compared with LH09. nd, non detected.

**TABLE S13.** Single point differences between parental (LH) and evolved strain LH03^a^.

|  |  |  | | |  |  | | |
| --- | --- | --- | --- | --- | --- | --- | --- | --- |
|  |  | **SNP frequency (%)** | | |  | **Effects^b^** | | |
|  |  |  |  |  |  |  |  |  |
| **Chr.** | **Position** | **LH** | **LH03** | **LH09** | **Affected Gene** | **Change** | **Variant type** | **Codon usage** |
| II | 168,941 | 2.9 | 20.0 | 0.0 | *RPL19B* | GTC>GTT | syn (V>V) | 0,21>0,39 |
| II | 168,950 | 4.4 | 21.4 | 4.3 | *RPL19B* | GGA>GGT | syn (G>G) | 0,12>0,47 |
| II | 168,959 | 2.9 | 16.7 | 4.5 | *RPL19B* | GTC>GTA | syn (V>V) | 0,21>0,21 |
| II | 169,058 | 2.5 | 17.1 | 4.6 | *RPL19B* | ACT>ACC | syn (V>V) | 0,35>0,22 |
| II | 169,067 | 2.2 | 15.6 | 4.1 | *RPL19B* | GCT>GCC | syn (A>A) | 0,38>0,22 |
| II | 170,308 | 0.0 | 19.1 | 0.0 |  | A>G | n.a. | n.a. |
| II | 170,310 | 0.0 | 16.3 | 0.0 |  | A>C | n.a. | n.a. |
| II | 320,417 | 2.8 | 22.9 | 6.6 | *CST26* | TGA>TAA | syn (STOP>STOP) | 0,29>0,48 |
| IV | 134,438 | 1.7 | 31.1 | 3.9 | *LYS20* | ATC>ATT | syn (I>I) | 0,26>0,46 |
| IV | 134,480 | 4.4 | 24.0 | 4.8 | *LYS20* | GCC>GCA | syn (A>A) | 0,22>0,29 |
| IV | 134,492 | 4.7 | 24.5 | 0.0 | *LYS20* | CAG>CAA | syn (Q>Q) | 0,31>0,69 |
| IV | 491,928 | 4.7 | 16.3 | 2.2 | *RPS11A* | GTT>GTC | syn (V>V) | 0,39>0,21 |
| IV | 492,018 | 4.0 | 23.8 | 3.1 | *RPS11A* | GAC>GAT | syn (D>D) | 0,35>0,65 |
| IV | 492,228 | 3.1 | 25.0 | 1.0 | *RPS11A* | GTC>GTT | syn (V>V) | 0,21>0,39 |
| IV | 492,231 | 4.1 | 21.6 | 1.0 | *RPS11A* | GCC>GCT | syn (A>A) | 0,22>0,38 |
| IV | 492,270 | 4.8 | 17.5 | 0.0 | *RPS11A* | GTT>GTC | syn (V>V) | 0,39>0,21 |
| IV | 492,291 | 4.0 | 16.7 | 0.0 | *RPS11A* | GCT>GCC | syn (A>A) | 0,38>0,22 |
| IV | 492,300 | 4.2 | 16.7 | 0.0 | *RPS11A* | GCT>GCC | syn (A>A) | 0,38>0,22 |
| IV | 492,306 | 4.3 | 16.7 | 0.0 | *RPS11A* | AAA>AAG | syn (K>K) | 0,58>0,42 |
| IV | 1,088,636 | 4.4 | 20.0 | 4.7 | *SSF2* | TTA>TTG | syn (L>L) | 0,28>0,29 |
| IV | 1,088,725 | 3.1 | 18.9 | 4.7 | *SSF2* | AGA>AAA | miss (R>K) | 0,48>0,58 |
| IV | 1,360,567 | 1.7 | 19.6 | 3.4 | *RPS18A* | GTT>GTC | syn (V>V) | 0,39>0,21 |
| IV | 1,360,573 | 1.8 | 17.4 | 4.3 | *RPS18A* | AAT>ATC | syn (T>T) | 0,46>0,26 |
| IV | 1,360,591 | 0.8 | 17.5 | 3.4 | *RPS18A* | CAC>CAT | syn (H>H) | 0,36>0,64 |
| IV | 1,360,597 | 1.7 | 23.8 | 2.8 | *RPS18A* | AAA>AAG | syn (K>K) | 0,58>0,42 |
| IV | 1,360,606 | 1.6 | 23.8 | 2.1 | *RPS18A* | GCT>GCC | syn (A>A) | 0,38>0,22 |
| IV | 1,360,615 | 1.7 | 25.0 | 2.3 | *RPS18A* | TTG>TTA | syn (L>L) | 0,29>0,28 |
| IV | 1,360,627 | 1.7 | 23.8 | 2.9 | *RPS18A* | AAT>AAC | syn (N>N) | 0,59>0,41 |
| IV | 1,360,633 | 1.7 | 26.2 | 3.0 | *RPS18A* | ATC>ATT | syn (I>I) | 0,26>0,46 |
| IV | 1,360,681 | 2.0 | 25.0 | 3.2 | *RPS18A* | AAA>AAG | syn (K>K) | 0,58>0,42 |
| IV | 1,360,711 | 1.9 | 22.5 | 3.6 | *RPS18A* | AAA>AAG | syn (K>K) | 0,58>0,42 |
| IV | 1,360,720 | 2.0 | 22.2 | 2.2 | *RPS18A* | GCT>GCC | syn (A>A) | 0,38>0,22 |
| IV | 1,360,723 | 1.9 | 19.4 | 3.0 | *RPS18A* | CAT>CAC | syn (H>H) | 0,64>0,36 |
| IV | 1,360,732 | 2.0 | 21.1 | 3.2 | *RPS18A* | ATC>ATT | syn (I>I) | 0,26>0,46 |
| IV | 1,360,750 | 2.2 | 19.1 | 3.6 | *RPS18A* | TTG>TTA | syn (L>L) | 0,29>0,28 |
| V | 326,002 | 0.0 | 17.5 | 2.0 |  | AT>A | n.a | n.a. |
| V | 397,296 | 2.9 | 15.2 | 3.2 | *RPL23B* | GTT>GTC | syn (V>V) | 0,39>0,21 |
| V | 397,302 | 3.4 | 17.1 | 3.5 | *RPL23B* | GCT>GCC | syn (A>A) | 0,38>0,22 |
| V | 397,347 | 3.9 | 19.4 | 5.8 | *RPL23B* | ATC>ATT | syn (I>I) | 0,26>0,46 |
| VI | 194,604 | 0.0 | 29.5 | 2.5 |  | TTA>T | n.a | n.a. |
| VII | 787,669 | 2.6 | 21.1 | 2.9 | *RPL24B* | GCT>GCC | syn (A>A) | 0,38>0,22 |
| IX | 177,481 | 0.0 | 21.2 | 0.0 |  | A>G | n.a | n.a. |
| IX | 245,697 | 1.6 | 22.7 | 0.5 |  | G>A | n.a | n.a. |
| IX | 317,839 | 0.0 | 23.5 | 7.0 | *RPL2B* | ACT>ACC | syn (T>T) | 0,35>0,22 |
| IX | 317,872 | 0.0 | 20.5 | 7.8 | *RPL2B* | GCT>GCC | syn (A>A) | 0,38>0,22 |
| IX | 317,884 | 0.0 | 20.5 | 7.8 | *RPL2B* | GCT>GCC | syn (A>A) | 0,38>0,22 |
| IX | 317,896 | 0.0 | 23.1 | 7.4 | *RPL2B* | ACC>ACT | syn (T>T) | 0,22>0,35 |
| XI | 68,106 | 3.6 | 20.4 | 2.3 |  | A>AAAAAAT | n.a | n.a. |
| XI | 68,113 | 3.9 | 23.5 | 2.4 |  | T>A | n.a | n.a. |
|  |  |  |  |  |  |  |  |  |
| **TABLE S13 (Continuation)** | | | | | | | | |
|  |  |  |  |  |  |  |  |  |
|  |  |  |  |  |  |  |  |  |
|  |  | **SNP frequency (%)** | | |  | **Effects^b^** | | |
|  |  |  | | |  |  | | |
| **Chr.** | **Position** | **LH** | **LH03** | **LH09** | **Affected Gene** | **Change** | **Variant type** | **Codon usage** |
| XI | 68,117 | 4.3 | 27.1 | 2.7 |  | G>A | n.a | n.a. |
| XI | 235,973 | 0.0 | 33.3 | 4.7 |  | G>GTA | n.a | n.a. |
| XII | 139,773 | 2.2 | 29.0 | 6.2 | *SPO75* | TGC>TGT | syn (C>C) | 0,37>0,63 |
| XII | 460,686 | 3.9 | 25.1 | 1.4 |  | A>A_6_GA_4_T | n.a | n.a. |
| XII | 460,720 | 0.0 | 16.2 | 1.4 |  | T>A | n.a | n.a. |
| XII | 460,722 | 4.9 | 21.3 | 4.1 |  | GTA>G: | n.a | n.a. |
| XIII | 8,370 | 4.8 | 28.1 | 2.6 | *COS3* | GTG>GAG | miss (V>E) | 0,19>0,29 |
| XIII | 8,373 | 4.8 | 23.3 | 2.5 | *COS3* | CTC>CCC | miss (L>P) | 0,06>0,15 |
| XIII | 37,749 | 0.0 | 17.7 | 3.6 | *YML116W-A* | T>0 | frameshift | n.a. |
| XIII | 550,539 | 4.8 | 20.5 | 3.0 | *RPL13B* | TAC>TAT | syn (Y>Y) | 0,44>0,56 |
| XIII | 550,674 | 2.9 | 17.5 | 1.7 | *RPL13B* | GCC>GCT | syn (A>A) | 0,22>0,38 |
| XIV | 181,308 | 4.6 | 17.1 | 3.9 |  | C>CA | n.a | n.a. |
| XIV | 258,275 | 0.0 | 21.9 | 0.0 |  | G>A | n.a | n.a. |
| XIV | 500,112 | 4.4 | 21.2 | 1.4 | *RPL9B* | ATC>ATT | syn (I>I) | 0,26>0,46 |
| XIV | 500,118 | 4.3 | 20.8 | 1.4 | *RPL9B* | TTA>TTG | syn (L>L) | 0,28>0,29 |
| XIV | 500,121 | 4.4 | 21.2 | 1.4 | *RPL9B* | TCT>TCA | syn (S>S) | 0,26>0,21 |
| XIV | 500,133 | 3.4 | 23.4 | 0.9 | *RPL9B* | GTT>GTC | syn (V>V) | 0,39>0,21 |
| XIV | 500,151 | 2.1 | 27.7 | 0.9 | *RPL9B* | AAT>AAC | syn (N>N) | 0,59>0,41 |
| XIV | 500,154 | 2.0 | 29.8 | 0.9 | *RPL9B* | GCC>GCT | syn (A>A) | 0,22>0,38 |
| XIV | 500,181 | 1.5 | 24.4 | 0.0 | *RPL9B* | GTT>GTC | syn (V>V) | 0,39>0,21 |
| XIV | 500,202 | 1.6 | 25.0 | 0.0 | *RPL9B* | AAG>AAA | syn (K>K) | 0,42>0,58 |
| XIV | 561,332 | 0.0 | 22.6 | 3.6 |  | G>A | n.a | n.a. |
| XV | 235,401 | 0.0 | 18.6 | 0.0 | *GAL11* | CAG>CAA | syn (Q>Q) | 0,31>0,69 |
| XV | 235,404 | 0.0 | 19.0 | 0.0 | *GAL11* | CAG>CAA | syn (Q>Q) | 0,31>0,69 |
| XV | 235,407 | 0.0 | 18.6 | 0.0 | *GAL11* | CAG>CAA | syn (Q>Q) | 0,31>0,69 |
| XV | 326,506 | 0.0 | 40.6 | 0.0 |  | T>A | n.a | n.a. |
| XV | 692,618 | 0.0 | 26.2 | 0.0 | *ULS1* | GTC>GTT | syn (V>V) | 0,21>0,39 |
| XVI | 436,481 | 4.9 | 38.3 | 3.5 |  | A>G | n.a | n.a. |
| XVI | 747,862 | 2.3 | 19.6 | 2.7 | *DBF20* | CA>C | frameshift | n.a. |

^a^ SNPs supported by **>**15% of the base calls for the position in the evolved strain and **<**5% of the calls in the parental strain. ^b^ syn, synonymous variant; miss, missense variant; n.a., non applicable.

**TABLE S14.** Single point differences between parental (LH) and evolved strain LH09^a^.

|  |  |  | | |  |  | | |
| --- | --- | --- | --- | --- | --- | --- | --- | --- |
|  |  | **SNP frequency (%)** | | |  | **Effects^b^** | | |
|  |  |  |  |  |  |  |  |  |
| **Chr.** | **Position** | **LH** | **LH03** | **LH09** | **Affected Gene** | **Change** | **Variant type** | **Codon usage** |
| IV | 383449 | 4.7 | 11.8 | 15.6 | *PRM7* | GAC>GAT | syn (D>D) | 0,35>0,65 |
| V | 455615 | 0.0 | 0.0 | 27.0 | *MAG1* | AGT>ATT | miss (S>I) | 0,16>0,46 |
| VI | 268205 | 0.0 | 5.9 | 23.5 |  | A>AAT | n.a. | n.a. |
| VIII | 146752 | 0.0 | 0.1 | 15.7 |  | G>A | n.a. | n.a. |
| XI | 146918 | 4.3 | 6.7 | 26.1 |  | C>T | n.a. | n.a. |
| XIII | 721996 | 0.0 | 0.0 | 29.6 | *YMR226C* | ATC>ACC | miss (I>T) | 0,26>0,22 |
| XV | 92645 | 0.0 | 0.0 | 41.7 |  | AT>A | n.a. | n.a. |
| XV | 108996 | 0.8 | 1.7 | 45.4 |  | T>C | n.a. | n.a. |
| XV | 108997 | 0.8 | 1.7 | 46.6 |  | CT>C | n.a. | n.a. |
| XV | 849793 | 0.0 | 0.0 | 20.8 | *RDL1* | TCG>TCC | syn (S>S) | 0,1>0,16 |

^a^ SNPs supported by **>**15% of the base calls for the position in the evolved strain and **<**5% of the calls in the parental strain. ^b^ syn, synonymous variant; miss, missense variant; n.a., non applicable.

**TABLE S15.** Single point differences with the parental strain (LH) common in both evolved strains (LH03 and LH09)^a^.

|  |  |  | | |  |  | | |
| --- | --- | --- | --- | --- | --- | --- | --- | --- |
|  |  | **SNP frequency (%)** | | |  | **Effects^b^** | | |
|  |  |  |  |  |  |  |  |  |
| **Chr.** | **Position** | **LH** | **LH03** | **LH09** | **Affected Gene** | **Change** | **Variant type** | **Codon usage** |
| II | 320502 | 4.5 | 18.6 | 21.7 | *CST26* | ATT>GAT | miss (N>D) | 0,59>0,65 |
| II | 586459 | 0.0 | 29.3 | 21.8 |  | G>GA | n.a | n.a |
| II | 586488 | 0.0 | 19.5 | 17.7 |  | A>T | n.a | n.a |
| III | 273011 | 2.6 | 25.7 | 39.6 |  | G>A | n.a | n.a |
| III | 63401 | 0.0 | 35.6 | 16.0 |  | CG> C | n.a | n.a |
| III | 63403 | 0.0 | 35.1 | 16.2 |  | A>C | n.a | n.a |
| IV | 501227 | 0.0 | 36.6 | 42.9 | *YDR029W* | CGT>CAT | miss (R>H) | 0,15>0,64 |
| V | 156147 | 0.0 | 43.2 | 25.6 |  | TC>T | n.a | n.a |
| V | 373393 | 0.0 | 29.2 | 57.7 |  | T>TA | n.a | n.a |
| V | 156159 | 2.7 | 44.4 | 26.3 |  | T>C | n.a | n.a |
| V | 156151 | 0.0 | 42.5 | 26.3 |  | G>A | n.a | n.a |
| V | 565610 | 4.4 | 25.8 | 19.7 |  | G>A | n.a | n.a |
| V | 565623 | 3.9 | 23.0 | 18.0 |  | C>T | n.a | n.a |
| VII | 483370 | 4.1 | 16.7 | 27.6 | *BRP1* | TTA>TTG | syn (L>L) | 0,28>0,29 |
| VII | 508794 | 3.0 | 32.8 | 24.4 |  | G>GTCT_4_ATCT_4_A | n.a | n.a |
| VIII | 365433 | 0.0 | 33.3 | 16.7 | *YHR131C* | GAT>GAC | syn (D>D) | 0,65>0,35 |
| X | 228720 | 4.8 | 32.6 | 31.2 |  | T>C | n.a | n.a |
| XI | 13740 | 1.5 | 58.2 | 46.9 |  | TC>T | n.a | n.a |
| XII | 937372 | 4.0 | 20.9 | 15.1 |  | TA>T | n.a | n.a |
| XII | 208656 | 2.8 | 26.8 | 19.3 |  | T>TAAGA | n.a | n.a |
| XII | 225364 | 0.9 | 49.4 | 20.3 |  | T>TA | n.a | n.a |
| XIII | 158979 | 2.9 | 17.4 | 16.1 |  | C>CT | n.a | n.a |
| XIII | 855378 | 3.6 | 30.0 | 61.2 |  | A>AT | n.a | n.a |
| XIV | 308657 | 0.0 | 66.7 | 68.1 |  | G>C | n.a | n.a |
| XIV | 308655 | 0.0 | 67.8 | 69.3 |  | CT>C | n.a | n.a |
| XIV | 628629 | 1.8 | 40.5 | 51.1 |  | A>AC | n.a | n.a |
| XV | 436844 | 0.0 | 23.3 | 26.1 |  | T>TA | n.a | n.a |

^a^ SNPs supported by **>**15% of the base calls for the position in the evolved strain and **<**5% of the calls in the parental strain. ^b^ syn, synonymous variant; miss, missense variant; n.a., non applicable.
